# Are Organic Falls Bridging Reduced Environments in the Deep Sea? - Results from Colonization Experiments in the Gulf of Cádiz

**DOI:** 10.1371/journal.pone.0076688

**Published:** 2013-10-02

**Authors:** Marina R. Cunha, Fábio L. Matos, Luciana Génio, Ana Hilário, Carlos J. Moura, Ascensão Ravara, Clara F. Rodrigues

**Affiliations:** 1 Departamento de Biologia and Centro de Estudos do Ambiente e do Mar, Universidade de Aveiro, Campus de Santiago, Aveiro, Portugal; 2 Departamento de Oceanografia e Pescas, Universidade dos Açores, Horta, Açores, Portugal; Northwest Fisheries Science Center, NOAA Fisheries, United States of America

## Abstract

Organic falls create localised patches of organic enrichment and disturbance where enhanced degradation is mediated by diversified microbial assemblages and specialized fauna. The view of organic falls as “stepping stones” for the colonization of deep-sea reducing environments has been often loosely used, but much remains to be proven concerning their capability to bridge dispersal among such environments. Aiming the clarification of this issue, we used an experimental approach to answer the following questions:

Are relatively small organic falls in the deep sea capable of sustaining taxonomically and trophically diverse assemblages over demographically relevant temporal scales?

Are there important depth- or site-related sources of variability for the composition and structure of these assemblages?

Is the proximity of other reducing environments influential for their colonization?

We analysed the taxonomical and trophic diversity patterns and partitioning (α- and β-diversity) of the macrofaunal assemblages recruited in small colonization devices with organic and inorganic substrata after 1-2 years of deployment on mud volcanoes of the Gulf of Cádiz. Our results show that small organic falls can sustain highly diverse and trophically coherent assemblages for time periods allowing growth to reproductive maturity, and successive generations of dominant species. The composition and structure of the assemblages showed variability consistent with their biogeographic and bathymetric contexts. However, the proximity of cold seeps had limited influence on the similarity between the assemblages of these two habitats and organic falls sustained a distinctive fauna with dominant substrate-specific taxa. We conclude that it is unlikely that small organic falls may regularly ensure population connectivity among cold seeps and vents. They may be a recurrent source of evolutionary candidates for the colonization of such ecosystems. However, there may be a critical size of organic fall to create the necessary intense and persistent reducing conditions for sustaining typical chemosymbiotic vent and seep organisms.

## Introduction

The interest in organisms associated with organic falls, and particularly sunken wood, was renewed by a comparative phylogenetic study [1] suggesting that vent and seep organisms may have recently diverged from faunas associated with organic falls. Ecological, phylogenetic and paleontological data [1,2,3] led to the hypothesis that organic falls provide important habitat islands for the persistence of species dependent on organic- and sulphide-rich conditions and could play a major role in the adaptation, and evolution of deep-sea chemoautotrophy-based assemblages. New issues have been raised recently regarding the prospect of a climate change-related increase in the exportation of terrigenous organic material to the deep sea and its potential impact on the future evolutionary ecology of deep-sea assemblages [4,5].

Important amounts of terrestrial plant materials and macroalgae debris are deposited frequently on continental slopes through physical or biological processes, primarily after extreme climatic events [6,7,8,9]. Plant remains are thus abundant in many areas: higher densities are likely to occur near estuaries, in sedimentary accumulation basins and in submarine canyons [8,10,11,12], but small amounts of organic material may be ubiquitous across depths and ecosystems. These small organic falls may significantly shorten the long distances (often over 1000s km) separating the known locations of hydrothermal vents, cold seeps or large and more or less predictable organic falls. An important ecological characteristic of deep-sea benthic communities is that they are highly food-limited and many aspects of their structure and function are strongly modulated by the rate and nature of the input of detrital organic material produced in the euphotic zone [13]. Organic falls such as marine-mammal carcasses and accumulations of wood and other plant remains are therefore thought to be fundamental to the nutritional ecology of the deep sea by the direct or indirect mobilization of organic carbon, the increased flux of reduced chemicals, and their potential role as nitrate sinks [5,14,15,16,17].

Organic falls create localised patches of organic enrichment and chemical or physical disturbance and are thought to contribute to beta diversity [2,16,17,18]. Animals use plant materials either as substrate, shelter, or food and an outstanding original and diverse fauna has been revealed by sampling natural sunken-wood habitats or by deploying colonization experiments containing different kinds of organic material [16,19,20,21,22,23]. Increased food availability in organic falls is associated with locally-enhanced degradation processes mediated by specialized (e.g. wood-boring xylophagainae bivalves) and/or opportunistic fauna and highly diversified microbial activity [4,16,24,25,26]. The state of degradation, and the duration and development of the ecological succession depend upon the immersion period, type (lability) of the organic material [16] and environmental conditions which may alter its physical and biochemical properties. Decay of plant remains begins with the degradation of cellulose (and also lignin in wood) by heterotrophic aerobic and anaerobic bacteria and fungi leading to the accumulation of by-products such as hydrogen, sulphide, and perhaps methane and to the decrease of oxygen [25,26]. The sulphide produced by the bacteria may then be used as an energy source for carbon dioxide fixation by chemosynthetic organisms [25,26]. It is at this stage that sunken wood free-living microbial communities may begin to resemble those from whale falls, vents, and seeps [27]. Within a few months opportunistic species and sulphide-tolerant microbial grazers may reach high densities [16]. Several organisms (e.g. molluscs, crustaceans) feed on wood which often requires a heterotrophic gut microflora providing the host with exoenzymes for metabolizing refractory plant material [24,28,29]. Some organisms (e.g. bathymodiolin bivalves) are chemosymbiotic and have close relatives in other reducing habitats [30].

Accumulated paleontological evidence [3] points to bathymodiolin mussels occurring contemporarily at seeps and at wood and whale falls, or earlier at seeps, contrary to the pattern predicted by Distel et al. [1]. There are also parsimony arguments supporting chemoautotrophy in a siboglinid ancestor and the secondary loss of chemoautotrophy in the boneworm *Osedax* [31]. In fact, the geologic history of reducing environments places vents and seeps as being older than wood and wood being older than whales. Based on the notion that many modern members of wood- fall assemblages were already present in the Late Cretaceous, Kiel and Goedert [3] predicted that the wood-fall ecosystem evolved during this time. But the modern whale-fall ecosystem may have evolved from an even older source of scattered reducing habitats: the abundant marine reptiles of the Jurassic and Triassic [32].

Despite the strong evolutionary linkage between biological communities inhabiting the different reducing environments, their biodiversity and biogeography remain relatively tattered. Deep-sea chemosynthesis-based ecosystems usually share the presence of reduced inorganic compounds (methane, hydrogen sulphide, hydrogen, or a combination of these) but they are highly heterogeneous in their geological or biological origin. Due to high local production, metazoans are often released from the extreme food limitation prevalent in the deep sea but instead, a variety of geochemical and microbial processes that impose different regimes of disturbance, chemical toxicity and physiological stress may modulate community structure [17]. Although the macrofaunal structure (family level) of hydrothermal vents, cold seeps and organic falls share some characteristics (e.g. low diversity and high dominance), broad-scale analysis suggests that chemosynthesis-based macrofaunal assemblages exhibit a low degree of similarity at the species level across systems [17]. However, these results have yet to be confronted with the recent perspective of a putative continuum of abiotic and biotic characteristics between hydrothermal vents and cold seeps [33,34,35]. Comparisons need to be constrained by a better knowledge on the intricate effects of water depth, oceanography and geologic setting on the biodiversity of reducing environments and their huge variability within and across biogeographic regions

Since the first references to the evolutionary implications of organic falls as “stepping stones” [2,36]; the concept has often been used loosely, sometimes passing the misleading idea of almost interchangeability of the fauna from organic falls and other reducing environments. Notwithstanding the role of organic falls in the adaptation and evolution of deep-sea chemoautotrophy-based assemblages [1] much remains to be proven concerning their capability to bridge dispersal among such assemblages and currently ensure population connectivity of their species. Our objective is to contribute to the clarification of this issue by using an experimental approach to answer the following straightforward questions:

1 Are relatively small and localized inputs of organic matter in the deep sea capable of sustaining taxonomically and trophically diverse assemblages over demographically relevant temporal scales?
2 Are there important depth- or site-related sources of variability for the composition and structure of the assemblages recruited in organic falls?
3 Is the proximity of other reducing environments influential for an increased similarity to the assemblages recruited in organic falls?

We propose to discuss these questions by analysing the taxonomic and trophic diversity patterns and partitioning (α- and β-diversity) of the macrofaunal assemblages recruited in small colonization devices deployed with organic and inorganic substrata on mud volcanoes of the Gulf of Cádiz and recovered after 1-2 years of immersion.

## Material and Methods

### Colonization devices

**Figure 1:**
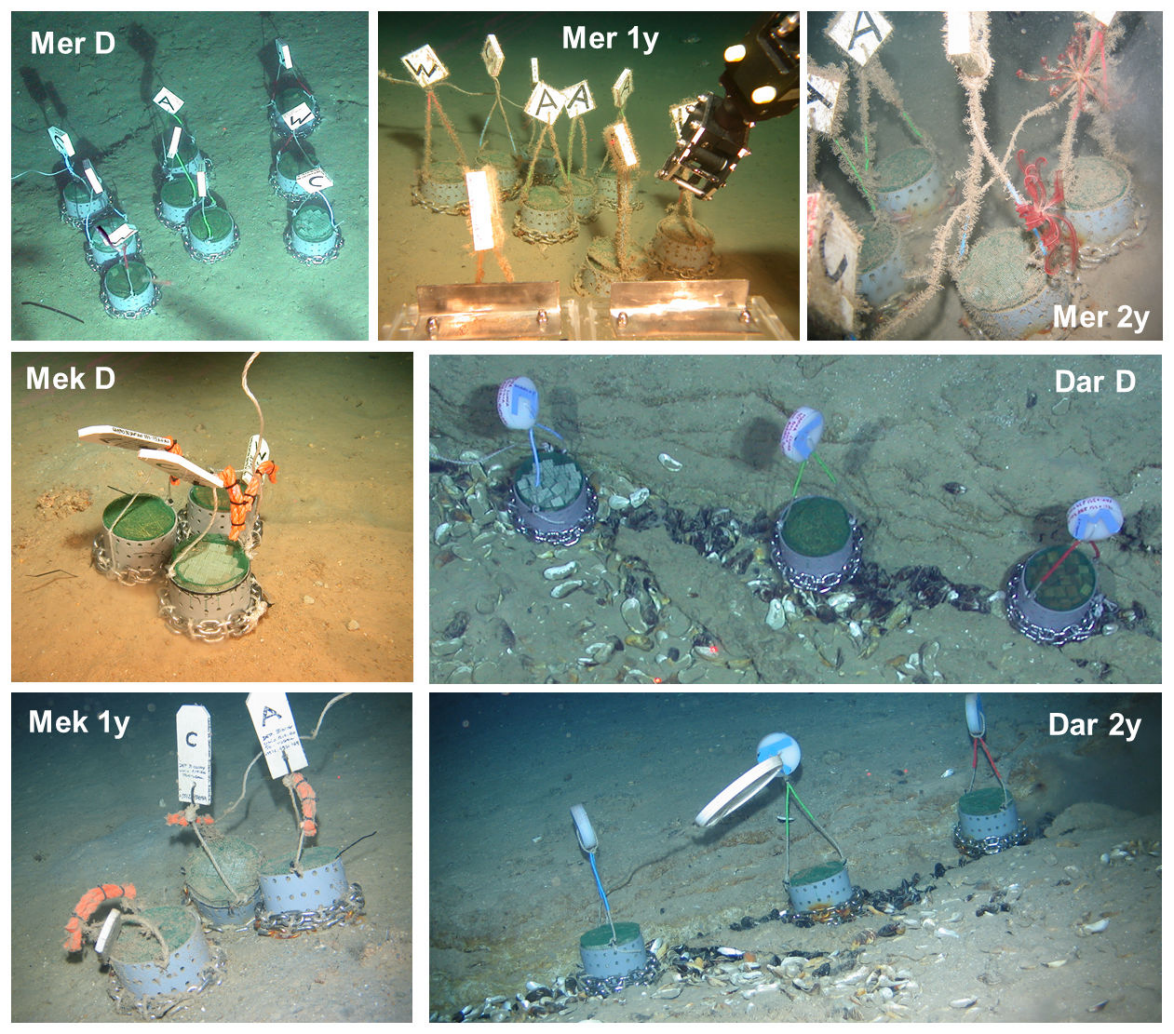
Aspect of the experimental colonization devices - CHEMECOLI (CHEMosynthetic Ecosystem COlonization by Larval Invertebrates), immediately after deployment (D) and after one (1y) and two (2y) years of immersion. Mer: Mercator MV; Mek: Meknès MV; Dar; Darwin (Photo credits: National Oceanography Centre, Southampton: Mer-D, Dar-D; MARUM, Universität Bremen, Mer-1y; Mek-D; Renard Center for Marine Research, Mer-2y, Mek-1y, Dar-2y).

In this study we used sets of standardized colonization experiments (CHEMECOLI - CHEMosynthetic Ecosystem COlonization by Larval Invertebrates, Figure 1) as described by Gaudron et al. [22]. Three types of substrate were used: dried alfalfa grass (more labile), Douglas fir wood cubes (less labile) and carbonate cubes (2x2x2cm) each totalling a volume of 1.539 dm^3^ inside a perforated PVC tube (14 cm diameter). Each type of organic or inorganic substrate was enclosed by a Nylon net of 2mm mesh which excludes large sized predators and allows the colonization by metazoan larvae or juveniles but not by the adults of most species.

### Deployment and recovery

The deployment and recovery of the in situ colonization experiments were made using a ROV (Remotely Operated Vehicle) during the JC10 cruise (ROV Isis, National Oceanography Centre, Southampton), the 64PE284 cruise (ROV Cherokee, MARUM, Bremen) and the B09/14 cruise (ROV Genesis, Renard Centre for Marine Geology, Gent). Sets of three CHEMECOLI, each with one type of substrate, were deployed in three mud volcanoes along a depth gradient (Table 1): Mercator located at 350m water depth in the El Arraiche Field, Meknès and Darwin located in the Carbonate Province at 700m and 1100m water depth, respectively (Additional information on the study sites is provided in Text S1 and Figure S1). The immersion time ranged from 290 to 631 days (Table 1) and the experiments were recovered in individual closed boxes with ambient water.

**Table 1:** Metadata for the deployment and recovery of the colonization experiments.

		**Site coordinates**				**Deployment**			**Recovery**			**DD**
**Site**	**Samples**	**Depth**	**Latitude**	**Longitude**		**Cruise-Dive**	**Date**		**Cruise/Dive**	**Date**		**(days)**
Mercator MV	Mer-1y	354 m	35°17.916’N	06°38.709’W		JC10-D28	2007.05.19		64PE284-D8/9	2008.03.02-3		290
Mercator MV	Mer-2y	354 m	35°17.916’N	06°38.709’W		JC10-D28	2007.05.19		B09/14b-D01	2009.05.19		631
Meknès MV	Mek-1y	698 m	34°59.091’N	07°04.424’W		64PE284-D07	2008.03.01		B09/14b-D03	2009.05.20		446
Darwin MV	Dar-2y	1100 m	35°23.523’N	07°11.513’W		JC10-D33	2007.05.21		B09/14b-D02	2009.05.19		629

DD: Deployment duration.

Diplomatic permits were obtained for the described field study from the Moroccan authorities for sampling in the Moroccan EEZ. The locations sampled are not privately-owned or protected in any way and the field studies did not involve endangered or protected species.

### Sample processing and taxonomic identification

After recovery the remaining amount of organic material was estimated and the different substrates were sub-sampled and processed for different purposes. Two thirds of each sample (the equivalent of 1 dm^3^ of the initially deployed substrate) were reserved for macrofaunal studies. The substrates were photographed and fixed directly (no washing or sieving) in 95% ethanol (one third) and 10% formalin (one third). The nets and other external components of the devices were washed and all fauna present was kept although not used for the data analysis. The sub-samples used for taxonomic studies were kept at room temperature. The animals were sorted under a stereomicroscope, identified to species level whenever possible, and deposited in the Biological Research Collection of Universidade de Aveiro where they remain available for further studies. Each species was allocated to a trophic guild (Table S1) defined by the feeding mode, food type, size and source, and life habit (trophic scheme adapted from [37]). The species list was compared to the existing check list of the macrofauna (including unpublished data) from the mud volcanoes of the Gulf of Cádiz [35] in order to detect new occurrences and species already reported from the local (individual mud volcano) or regional fauna (all mud volcanoes and adjacent cold-water coral and authigenic carbonate habitats).

### Data analysis

Species richness, Shannon-Wiener diversity index (H’), Pielou evenness index (J’), Hurlbert expected species richness (ES_(n)_), and k-dominance curves [38,39,40] were calculated using the community analysis PRIMER v6 software [41] which was also used for multivariate analyses. The abundance data were first organized into a sample vs species matrix and non-metric multidimensional scaling (nMDS) ordination was performed using the Bray and Curtis similarity measure, after square root transformation. An analysis of similarities by randomization/permutation tests (One-way ANOSIM) was performed on the MDS results to assess for differences in the assemblages putatively related to: i) location (Mercator in the El Arraiche Field, Meknès and Darwin in the Carbonate Province); ii) substrate type (wood, alfalfa, carbonate); iii) immersion period (1 year: 290-446 days; 2 years: 629-631 days). SIMPER analysis (Similarity Percentages – species contributions) was performed to assess the percentage contributions of each species to the similarity within, and dissimilarity between groups of samples.

For the analysis of β-diversity we used a dissimilarity matrix based on the Bray and Curtis measure after square root transformation. In order to describe different contributions for the overall β-diversity, the pairwise comparison values (pwc) were grouped into different categories: i) pwc between samples of the same substrate type within the same region; ii) pwc between samples of different substrate types within the same region; iii) pwc between samples of the same substrate type in different regions; iv) pwc between samples of different substrate types and different regions.

Diversity partitioning was assessed for species richness (S), Hurlbert’s expected number of species (ES_(30)_) and Shannon-Wiener index (H’) and their equivalents for trophic diversity. The total diversity (γ = α+ β) is partitioned into the average diversity within samples (α) and among samples (β) and therefore β-diversity can be estimated by β = γ -α [42,43]. To extend the partition across multiple scales (β_1_: within substrate type; β_2_: between substrate types; β_3_: between MV fields), the smallest sample unit for level 1 are individual samples, while for the upper levels sampling units are formed by pooling together the appropriate groups of nested samples. The diversity components are calculated as β_m_ = γ -α_m_ at the highest level and β_i_ = α_i+1_ -α_i_ for each lower level. The additive partition of diversity is γ = α_1_ + β_1_ + β_2_ + … + β_m_.

The total diversity can therefore be expressed as the percentage contributions of diversity in each hierarchical level [44]. Partitioning was carried out by weighting each sample according to its respective abundance. Values of α_i_ were therefore calculated has a weighted average (according to the abundance of samples pooled in each level).

## Results

### Composition and structure of the recruited assemblages

A total of 8497 individuals was recovered in the substrata enclosed by the 2mm mesh net and ascribed to 111 different species. Molluscs were the most abundant group (53.5% of the total number of individuals) followed by crustaceans (29.9%) and polychaetes (15.3%). However the latter were the most species rich with 46 species while molluscs and crustaceans were represented by only 24 species each. Additionally, 141 species were recovered from the external components of the colonization devices totalling 171 different species identified from these experiments (Table S1). All analyses below were performed using only the fauna enclosed by the net and refer to a standardised volume equivalent to 1dm^3^ of initially deployed substrate.

#### Substrate type

**Figure 2:**
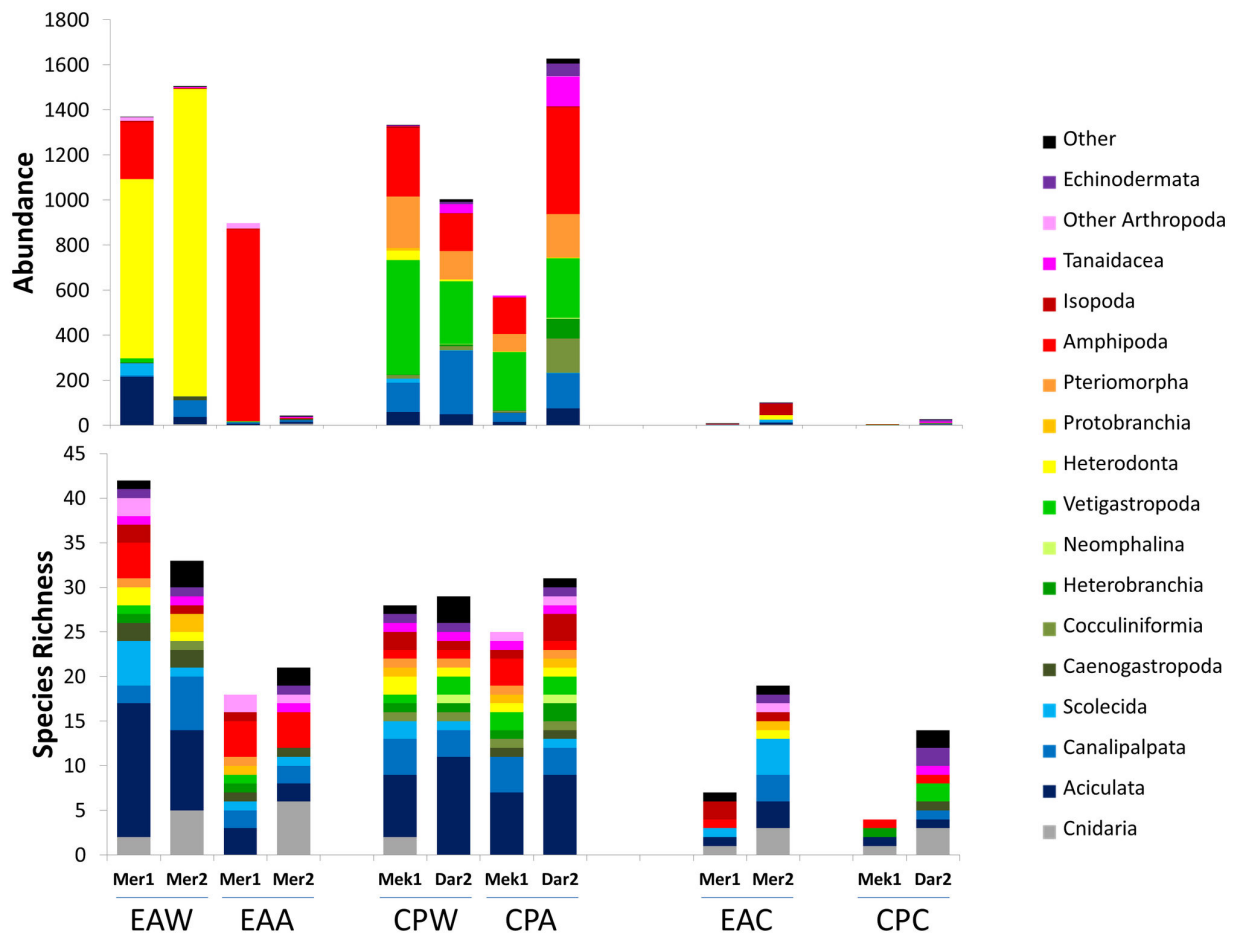
Species richness (bottom) and abundance (top) of the assemblages recruited in the different experiments. Numbers refer to 1 dm^3^ of initially deployed substrate. EA: El Arraiche field; CP: Carbonate Province; W: wood; A: alfalfa; C: carbonate; Mer: Mercator MV; Mek: Meknès MV; Dar: Darwin MV; 1: one year deployment; 2: 2 years deployment.

**Figure 3:**
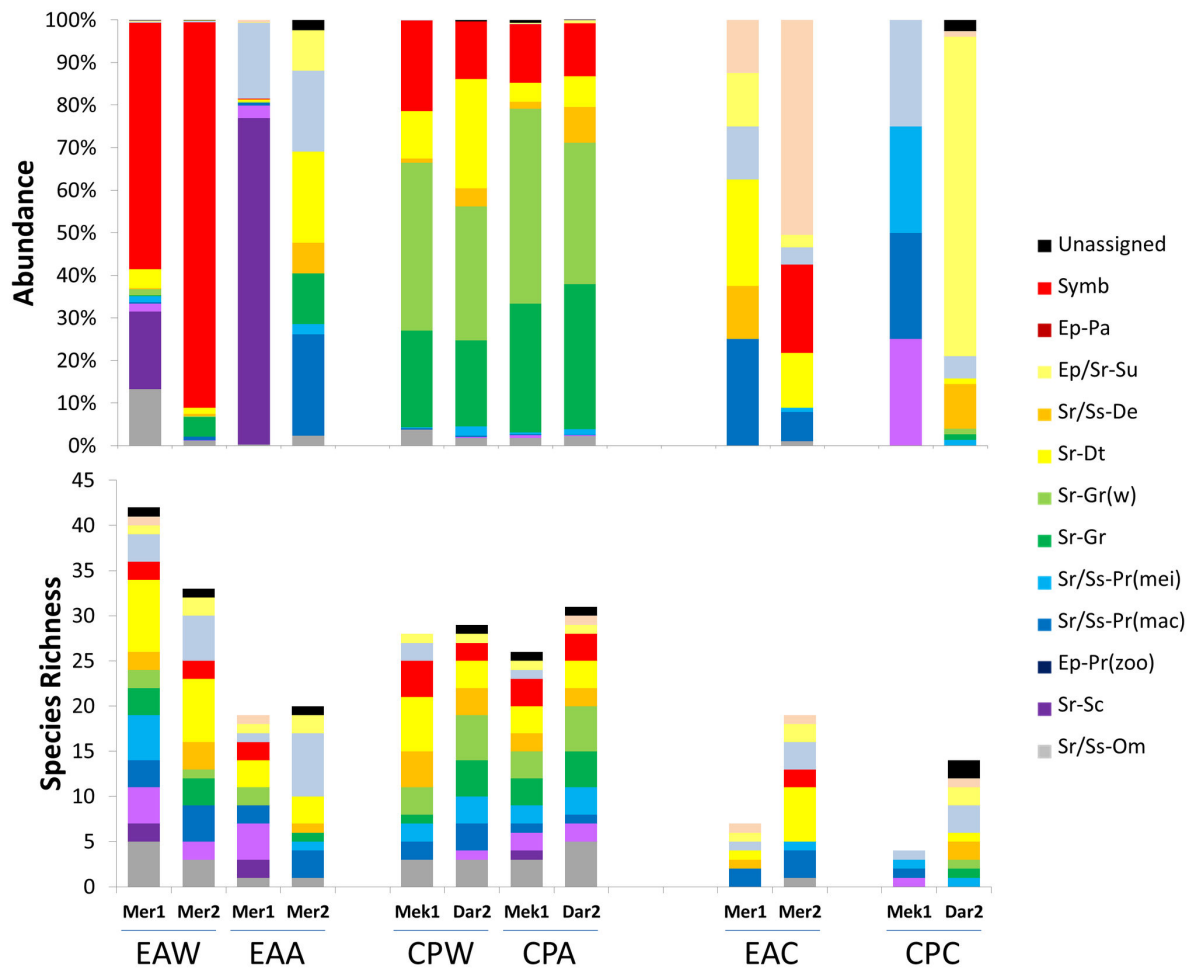
Trophic structure of the assemblages recruited in the different experiments: species richness and abundance of the different trophic groups. Symb: symbiotic; Ep: epibenthic feeding; Sr: surface feeding; Ss: sub-surface feeding; Pa: sectorial parasites; Su: suspension feeders; De: deposit feeders; Dt: detritivores; Gr: bacterial grazers; Gr(w): wood-specialist bacterial grazers; Pr(mei): predators feeding on meiofauna; Pr(mac): predators feeding on macrofauna; Pr(zoo): predators feeding on zooplankton; Sc: scavengers; Om: Omnivores (detailed information in Table 1). EA: El Arraiche field; CP: Carbonate Province; W: wood; A: alfalfa; C: carbonate; Mer: Mercator MV; Mek: Meknès MV; Dar: Darwin MV; 1: one year deployment; 2: 2 years deployment.

Overall organic substrata yielded much greater average abundances (wood: 1303 ind.dm^3^ ± 106.4; alfalfa: 786 ind.dm^3^ ± 331.3) and number of species (wood: 28-42; alfalfa: 18-31) than the inorganic ones (carbonate: 35 ind.dm^3^ ± 22.3; 4-19 species) with the highest variability recorded in the alfalfa assemblages (Table 2; Figure 2). The wood assemblages always showed relatively low evenness (J’: 0.158-0.651) and were highly dominated by the wood-boring bivalve *Xylophaga dorsalis* (up to 90% of the total abundance in Mercator MV) or by vetigastropod and amphipod bacterial grazers (*Copulabyssia* sp. and *Seba aloe* in Meknès and Darwin MVs) while the alfalfa assemblages showed higher variability in dominance, and in trophic structure (Table 2, Figures 2 and 3). The carbonate assemblages were formed by a very low number of individuals showing high evenness (J’: 0.606-1.000) but no clear trend in the dominant species. There was however preponderance for a fauna (e.g. hydrozoans, ophiuroids) relying upon epibenthic food sources. The significance of differences related to substrate type is supported by the ANOSIM results (Global R: 0.294; P: 3.3%). The subsequent pairwise comparisons also show low P values for differences between wood and carbonate assemblages and between alfalfa and carbonate although they are statistically significant only in the first case (Table 3). The dominant species in the organic substrata (the bivalves *X. dorsalis*, and *Idas modiolaeformis*, the vetigastropod *Copulabyssia* sp., the amphipods *S. aloe* and *Orchomene grimaldii* and the polychaete *Mellinopsis* sp.) are the main contributors for the dissimilarity between the assemblages of different substrate types retrieved by the SIMPER analysis (Table S2).

**Table 2:** Abundance and biodiversity data for individual and pooled samples.

Sample	n	A	SE	S	H’	J’	k1	First dominant	ES_(30)_	ES_(100)_
		(ind.dm^-3^)					(%)			
**Mer1W**	1	1369	—	42	1.54	0.413	57.9	*Xylophaga dorsalis*	6.7	11.6
**Mer2W**	1	1506	—	33	0.55	0.158	90.4	*Xylophaga dorsalis*	3.4	6.9
**Mer1A**	1	897	—	18	0.99	0.344	70.9	*Orchomene grimaldii*	4.3	6.9
**Mer2A**	1	43	—	21	2.72	0.894	16.3	*Melinnopsis* sp.	16.5	—
**Mek1W**	1	1332	—	28	1.82	0.545	38.1	*Copulabyssia* sp.	7.1	11.7
**Dar2W**	1	1004	—	29	2.19	0.651	27.6	*Copulabyssia* sp.	9.1	14.3
**Mek1A**	1	577	—	25	1.66	0.515	44.0	*Copulabyssia* sp.	6.7	11.8
**Dar2A**	1	1628	—	31	2.29	0.668	29.1	*Seba aloe*	10.1	14.9
**Mer1C**	1	9	—	7	1.89	0.971	22.2	Capitellidae sp05	—	—
**Mer2C**	1	100	—	19	1.79	0.606	51.0	*Gnathia* sp.	9.0	19.0
**Mek1C**	1	4	—	4	1.39	1.000	40.0	*Leocrates atlanticus*	—	—
**Dar2C**	1	26	—	14	2.27	0.859	26.9	*Mesotanais pinguiculus*	—	—
**EAW**	2	1438	68.5	60	1.20	0.292	74.9	*Xylophaga dorsalis*	5.9	11.5
**EAA**	2	470	427.0	36	1.24	0.347	67.7	*Orchomene grimaldii*	5.5	10.4
**CPW**	2	1168	164.0	42	2.08	0.557	33.6	*Copulabyssia* sp.	8.6	14.7
**CPA**	2	1103	525.5	41	2.22	0.597	28.6	*Seba aloe*	9.6	14.9
**EAC**	2	54.5	45.5	21	1.95	0.640	47.7	*Gnathia* sp.	9.9	20.0
**CPC**	2	15	11.0	17	2.50	0.881	22.6	*Mesotanais pinguiculus*	17.0	17.0
**W**	4	1303	106.4	74	2.20	0.511			9.8	17.2
**A**	4	786	331.3	66	2.51	0.599			11.1	18.3
**C**	4	35	22.3	35	2.53	0.712			13.3	28.5
**EA**	6	654	282.4	78	1.68	0.386			7.3	14.5
**CP**	6	762	275.9	69	2.25	0.532			9.6	16.1
**All**	12	708	188.9	111	2.60	0.553			11.5	20.6

n number of samples pooled; A: abundance; SE: standard error; S: species richness; H’: Shanon-Wienner diversity; J’Pielou’s evenness; k1: abundance contribution of the first dominant species; ES_(30)_ and ES_(100):_ Hulbert’s expected number of species per 30 and 100 individuals, respectively; Mer: Mercator MV; Mek: Meknès MV; Dar: Darwin MV; 1: 1-year immersion; 2-2-year immersion; W: wood; A: alfalfa; C: carbonate; EA: El Arraiche Field; CP: Carbonate Province.

**Table 3:** Results of the ANOSIM one-way analysis for global and pairwise tests for location (ANOSIM test 1), substrate type (ANOSIM test 2) and duration of the immersion period (ANOSIM test 3).

	**Sample statistic**	**Permutations used**	**Significant Statistics**	**Significance level**
**ANOSIM test 1**				
**Global test**				
Mud volcano	0.369	9240 (a)	180	1.9%*
**Pairwise tests**				
Mercator *vs* Meknès	0.481	84 (a)	2	2.4%*
Mercator *vs* Darwin	0.543	84 (a)	1	1.2%*
Meknès *vs* Darwin	0.000	10 (a)	5	50.0% ns
**ANOSIM test 2**				
**Global tests**				
Substrate	0.294	5775 (a)	191	3.3%*
**Pairwise tests**				
Wood *vs* Alfalfa	-0.031	35 (a)	23	65.7% ns
Wood *vs* Carbonate	0.589	35 (a)	1	2.9%*
Alfalfa *vs* Carbonate	0.328	35 (a)	4	11.4% ns
**ANOSIM test 3**				
**Global test**				
1 year *vs* 2 years	-0.035	462 (a)	250	54.1% ns

(a) all possible permutations; * significant; ns: not significant.

#### Site-related differences

**Figure 4:**
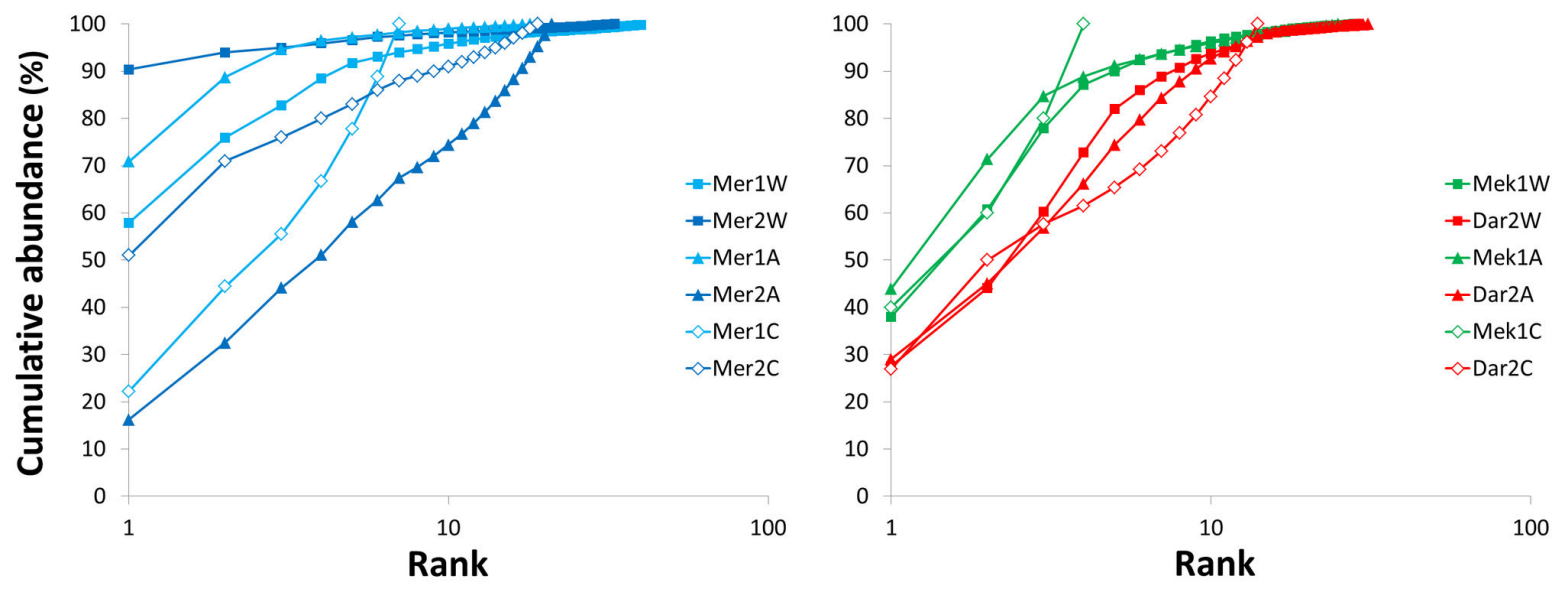
k-dominance curves of the assemblages recruited in the different experiments deployed in the El Arraiche field (left) and the Carbonate Province (right). Mer: Mercator MV; Mek: Meknès MV; Dar: Darwin MV; 1: one year deployment; 2: 2 years deployment; W: wood; A: alfalfa; C: carbonate.

The most striking feature of site-related patterns is that the recruited assemblages in Mercator MV (El Arraiche Field) show much higher variability than the two deeper mud volcanoes combined (Meknès and Darwin from the Carbonate Province). This applies to all abundance, diversity and evenness indicators (Table 2) and to the taxonomic and trophic structure of the assemblages from organic substrata (Figures 2 and 3) which are very similar in Meknès and Darwin MVs, whether they are from alfalfa or from wood. Also the k-dominance curves (Figure 4) show variability in Mercator MV and consistency in Meknès and Darwin MVs. In the Carbonate Province mud volcanoes the k-dominance curves are all overlapping (including the one representing carbonate assemblages) with a 10% distance between Darwin and Meknès assemblages. Site-related differences are statistically supported by the ANOSIM Global test (R: 0.369; P: 1.9%) and the subsequent pairwise tests also confirm the differences between Mercator MV and the two Carbonate Province mud volcanoes as well as the similarity of the latter (Table 3). Again, changes in the abundance of dominant species (*X. dorsalis* and *O. grimaldii* in Mercator and *Copulabyssia* sp., *S.* aloe, *I. modiolaeformis* and *Mellinopsis* sp. in the Carbonate Province) are the main contributors to explain site-related differences in the recruited assemblages (SIMPER analysis, Table S3).

#### Duration of the immersion period

Colonization rates of the carbonate substrate were very low despite a 2- to 3-fold increase in the number of species and a 6- to 10 fold increase in the abundance from one to two years of immersion (Table 2, Figure 2). In the Carbonate Province the assemblages from organic substrata showed little variation in composition and structure irrespective of the duration of the immersion period: a slightly higher number of species was recorded in Darwin MV (2 years) than in Meknès MV (1 year); abundance in alfalfa was higher in Darwin MV than in Meknès MV but the opposite situation was observed in the colonization of wood. The most drastic changes were observed in the colonization of alfalfa in Mercator MV. During the first year this substrate was heavily colonized by small amphipods: scavenger lysianassids (*O. grimaldii*) which are known to feed opportunistically on phytodetritus, and predator calliopids (*Leptamphopus* sp122). After two years the majority (ca. 90%) of the substrate was consumed and could no longer sustain these amphipods, leading to a sharp decrease in abundance and to a trophic composition much closer to the one found in the inorganic substrata (Figures 2 and 3). The wood substrate in Mercator MV was also considerably degraded by the action of the wood-boring bivalves *X. dorsalis*. After 2 years ca. 60% of the substrate was consumed and the assemblage tended to a higher taxonomical and trophic dominance. The low colonization by xylophagainae bivalves and lysianassid amphipods may explain the low level of degradation of the organic substrata in the deeper mud volcanoes (10-30% of the material was consumed). Changes in the composition and structure of the assemblages in relation to the duration of the immersion period were not consistent across different substrates or study regions and consequently the results of the ANOSIM test (Table 3) were not statistically significant. High numbers of recently settled recruits of limpets (*Coccopigya* sp. and *Copulabyssia* sp.) and bivalves (*X. dorsalis* and *I. modiolaeformis*) were observed. The permanence of the organic substrata over the experimental period of 2 years allowed the establishment of demographically viable populations of the dominant species - ovigerous females were frequent among amphipod populations, large xylophagainae bivalves included adult females with the associated dwarf males and mytilid bivalves were sexually mature (A. Hilário pers. observ. from histological slides).

#### Influence of the background fauna

**Figure 5:**
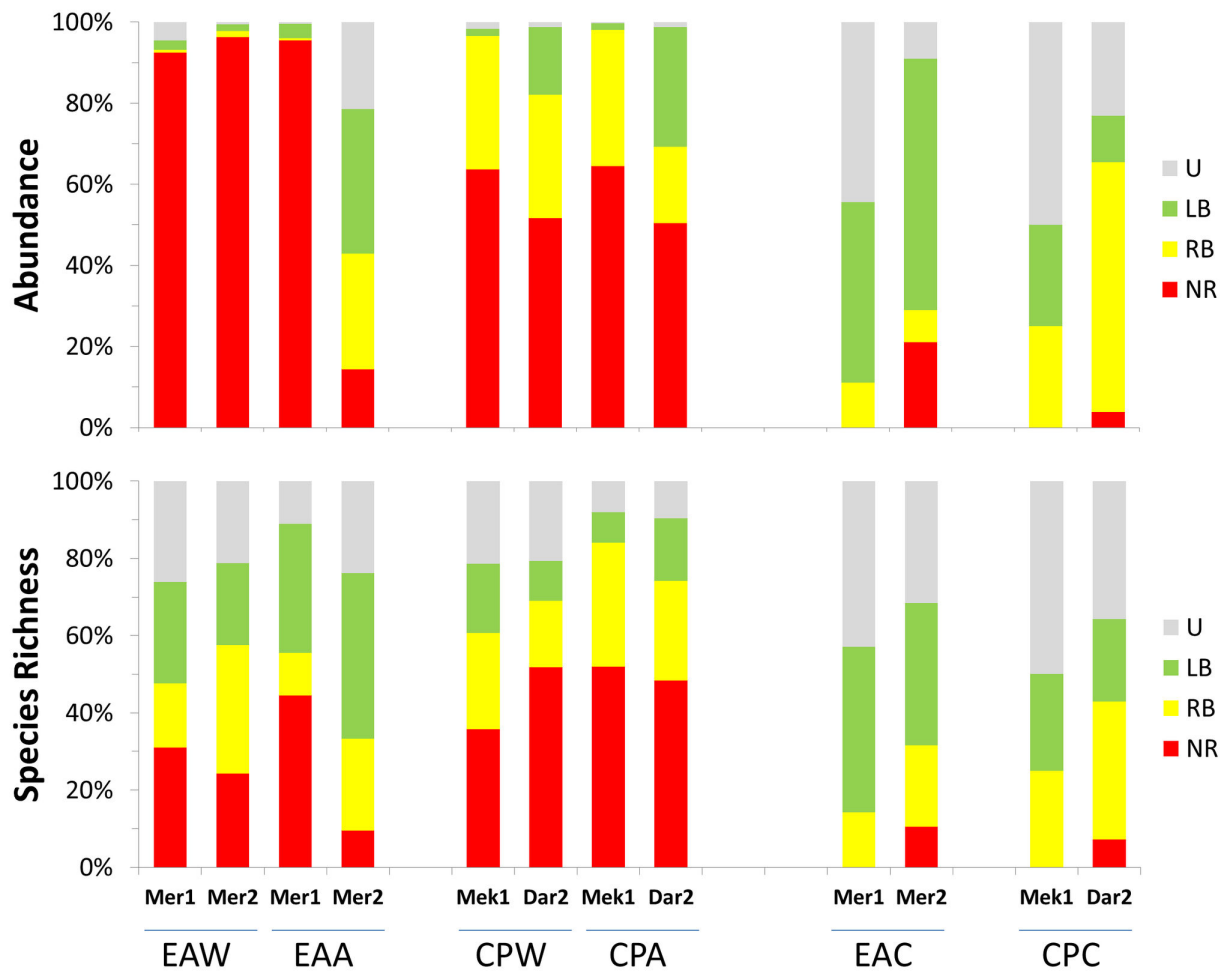
Relative contributions of background taxa and new occurrences to the species richness (bottom) and abundance (top) of the assemblages recruited in the different experiments. EA: El Arraiche field; CP: Carbonate Province; W: wood; A: alfalfa; C: carbonate; Mer: Mercator MV; Mek: Meknès MV; Dar: Darwin MV; 1: one year deployment; 2: 2 years deployment. U: unassigned; LB: local background; RB: regional background; NR: new records (detailed information in Table 1).

Overall 25% of the recruited species in the colonization experiments had not yet been reported from previous sampling in the study region. The new occurrences (Table S1) were mainly omnivore polychaetes (including a complex of *Ophryotrocha* species) and amphipods (small lysianassids), bacterial grazers (cocculinid gastropods, vetigastropods, the polychaete *Raricirrus beryli*), symbiotic xylophagainae (*X. dorsalis*, *Xyloredo* sp.) and mytilid bivalves (*I. modiolaeformis*), and a few predator and scavenger species (polychaetes, gastropods and lysianassid amphipods). The trophic groups that showed the lowest contributions from background fauna (Table S1) were omnivorous (25%), symbiotic (40%), grazers (40%), scavengers (50%). There were no new occurrences in the carbonate substrata after 1 year and only very few individuals of 1-2 species after 2 years. In contrast the organic substrata in Meknès and Darwin MVs recruited up to 15 new occurrences each, accounting for 36-52% of the species and 50-65% of the abundance. In Mercator MV new occurrences in the organic substrata were represented by fewer species (24-45%) but accounted for over 90% of the abundance. The exception was the alfalfa substrate after two years of immersion which showed a much higher relative contribution of the background fauna approaching the composition observed in the inorganic substrate (Figure 5).

#### Relevance of chemosymbiosis

Mercator MV was scarcely colonized by chemosymbiotic species (less than 1% of total abundance) independently of the substrate. In Meknès and Darwin MVs the abundance of chemosymbiotic species was considerable higher. Solemyidae were represented by only a few very small recruits but *Idas modiolaeformis* reached 12% of the total abundance in both organic substrates at Darwin MV and slightly higher values at Meknès MV (17 and 13% in wood and alfalfa, respectively). The presence of chemosymbiotic bivalves (Mytilidae and Solemyidae) was therefore recorded in organic substrates at the three mud volcanoes and while solemyids are part of the known local mud volcano fauna *I. modiolaeformis* is a new occurrence for this study area.

Despite the high contribution of the mud volcanoes’ heterotrophic fauna for the colonization of the deployed substrata the recruitment of local chemosymbiotic fauna was extraneous. Frenulate siboglinids are represented by several species in all studied sites but only one specimen occupying a 2cm long tube was found in the alfalfa sample from Meknès MV. Solemyid bivalves are known to occur in the three mud volcanoes (*Solemya* sp. in Mercator and *Acharax* sp. in Meknès and Darwin) and only a few specimens were found in the organic substrata. With the exception of one specimen found in alfalfa (Mer1), the solemyid bivalves (up to 13 ind. in Mek1-Alfalfa) were recently settled recruits with few or no signs of shell growth. Although Darwin MV sustains a living population of “*Bathymodiolus*” *mauritanicus*, molecular analyses made to a random selection of mytilid specimens recruited in the deployed substrata matched them all to *Idas modiolaeformis* [45].

### Diversity partitioning

**Figure 6:**
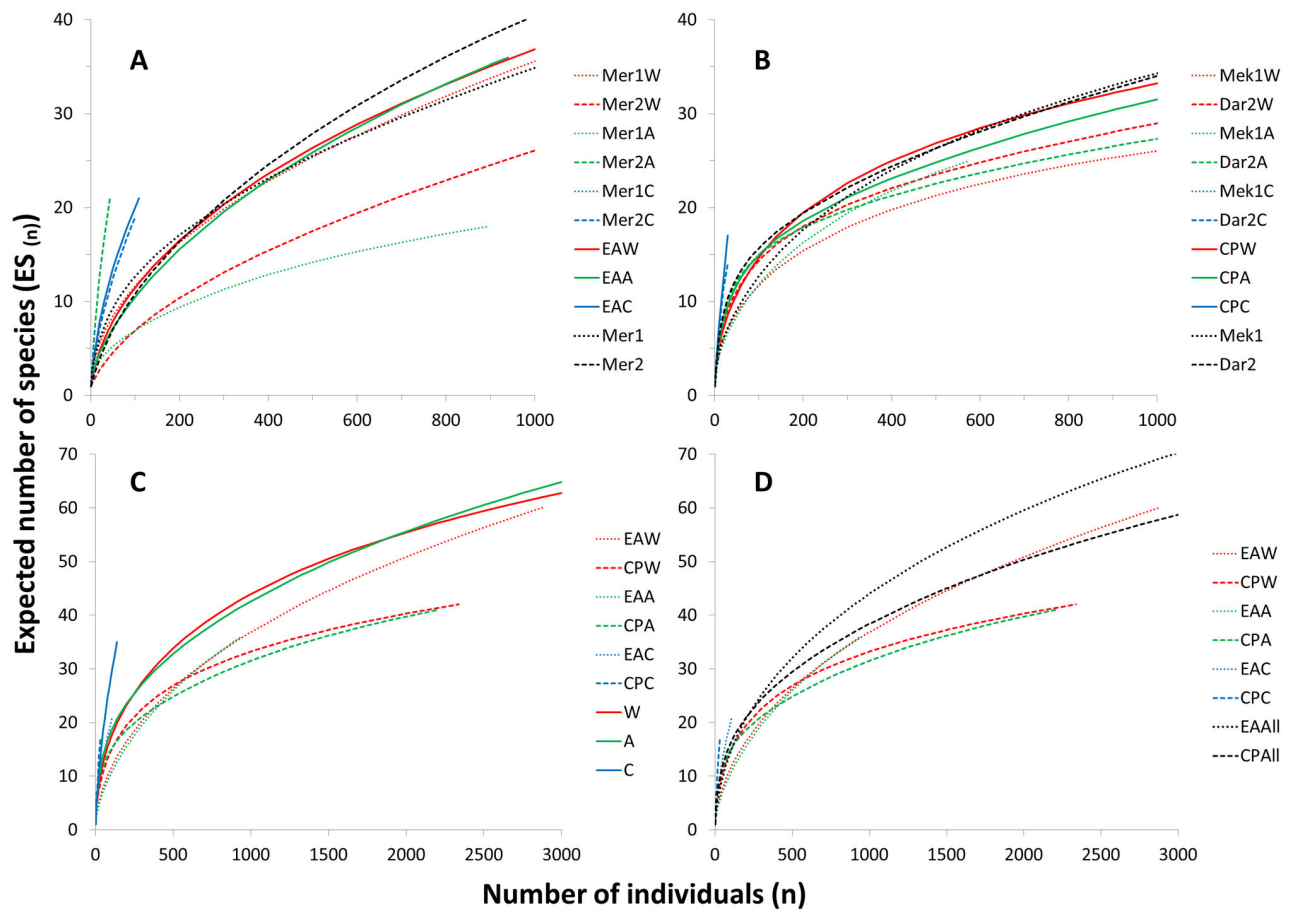
Comparison of rarefaction curves (Hurlbert’s expected number of species) for the assemblages assemblages recruited in the different experiments deployed in the El Arraiche field (A) and the Carbonate Province (B), pooled by substrate type (C) and pooled by sub-region (D). EA: El Arraiche field; CP: Carbonate Province; W: wood; A: alfalfa; C: carbonate; Mer: Mercator MV; Mek: Meknès MV; Dar: Darwin MV; 1: one year deployment; 2: 2 years deployment.

Patterns of α-diversity are illustrated by the rarefaction curves (Figure 6) which confirm the high variability of the assemblages in Mercator MV contrasting with the consistency in the assemblages from the two mud volcanoes of the Carbonate Province (Figure 6 A-B). When the samples are pooled per substrate type, the rarefaction curves for wood and alfalfa colonization are almost completely overlapping while the carbonate assemblage shows higher biodiversity (steeper slope) despite the low rate of colonization (Figure 6 C). The curves for the samples pooled by region are initially steep and overlapping; the biodiversity is slightly higher in the Carbonate Province for low numbers of individuals (n) but the rarefaction curve saturates more rapidly diverging (after n=200 ind., Figure 6 D) towards lower ES values than in Mercator MV (El Arraiche field).

**Figure 7:**
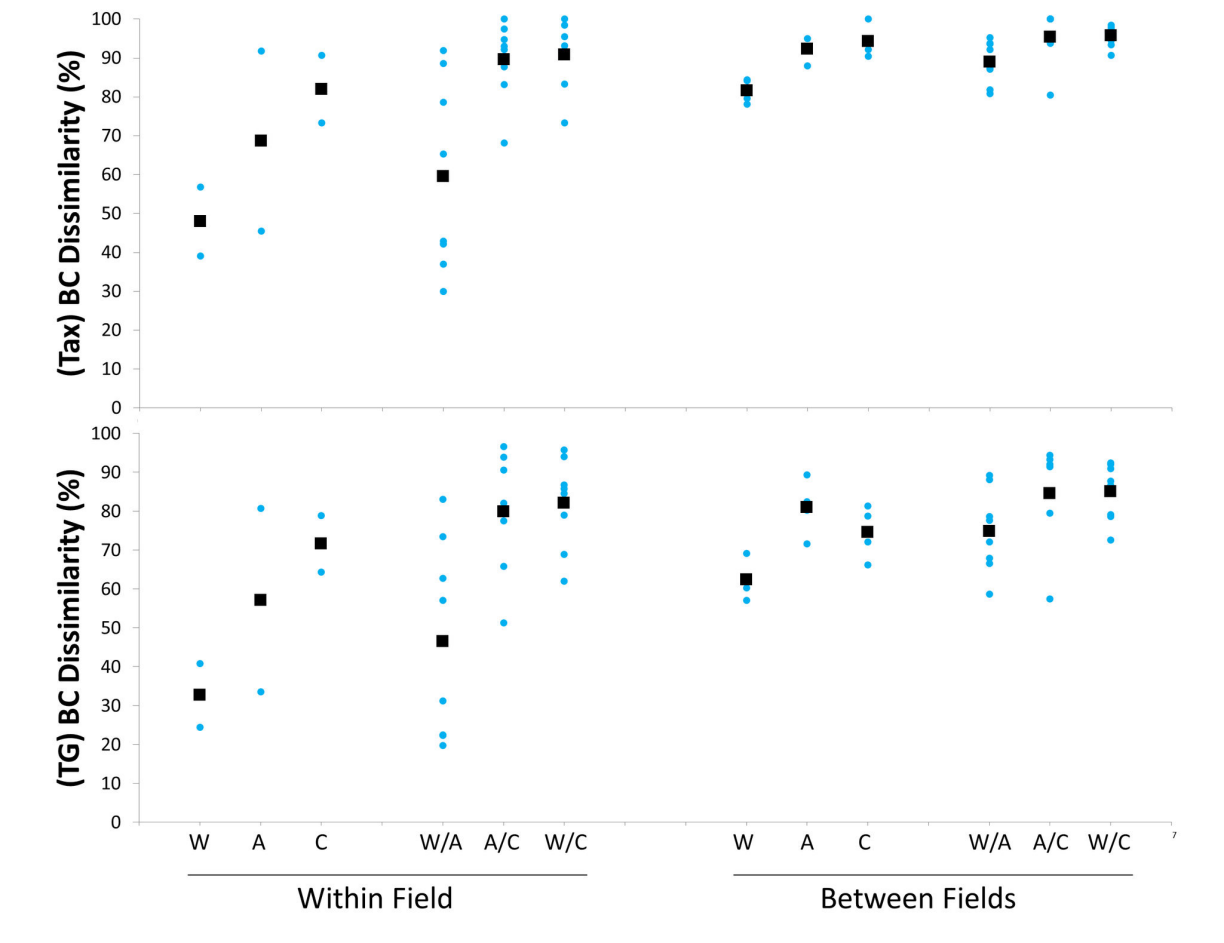
β-diversity analysis for species (top) and trophic groups (bottom) based on Bray-Curtis dissimilarity between all possible pairs of individual samples. Blue dots show the value of each pairwise comparison and black squares are the averages for each category. W: wood; A: alfalfa; C: carbonate.

The observed β-diversity (Figure 7) was lowest within assemblages from organic substrates deployed in the same region (average Bray and Curtis dissimilarity: 48-69%) and generally higher (81-96%) between assemblages from different regions. Nevertheless, comparisons between organic and inorganic substrata also yielded high dissimilarity values even within the same region (90-91%). The β-diversity patterns in taxonomic and trophic diversity were similar but with lower values (about 10-20% lower) in the latter.

**Figure 8:**
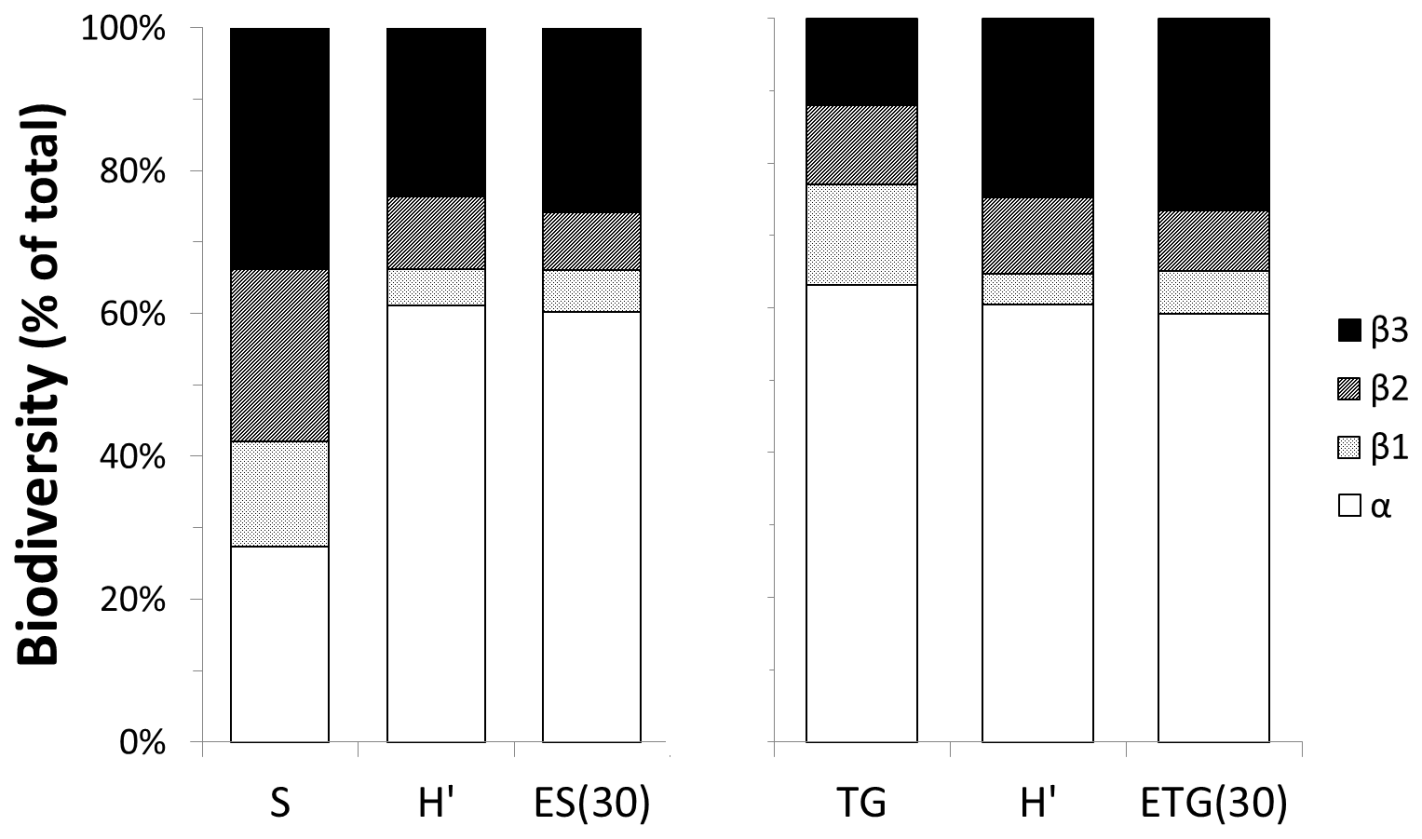
Partition of taxonomic (left) and trophic (right) diversity for different indices. S: number of species; H’: Shannon-Wiener diversity (ln-based); ES(30): Hurlbert expected number of species per 30 individuals; TG: number of trophic groups; ETG(30) Hurlbert expected number of trophic groups per 30 individuals; β_1_: β-diversity within same substrate and same sub-region; β_2_: β-diversity between different substrata in the same sub-region; β_3_: β-diversity between sub-regions.

Partitioning of diversity (Figure 8) is also similar in the taxonomic and trophic structure of the assemblages except for the number of species (S) and trophic guilds (TG). The contribution of α-diversity is rather low in terms on number of species and much higher in terms on number of trophic guilds meaning that even at the sample scale a relatively large number of different trophic groups is represented, and ensured, by a limited array of different species (i.e. there is a trophic coherence of the assemblages on a very small scale). The β-diversity associated with differences in substrate type (β_2_) and region (β_3_) is much higher for S than for TG because different species in different substrata and regions cover the same range of trophic guilds. For the indices related to community structure (H’ and ES_(30_)/ETG_(30)_) the diversity partition is similar for species and trophic guilds (differences of about 1%) because the effect of the few dominant species is mirrored by the effect of dominant trophic guilds. In the case of H’ and ES_(30)_, α-diversity makes most of the contribution to the global observed diversity suggesting some structural integrity (both taxonomic and trophic) of the assemblages at the small spatial scale (on average, about 60% of the total structural diversity is represented in each individual sample). Also important is the diversity explained by differences in H’ and ES_(30)_ between the two regions (β_3_: ca. 25%) which is higher than the one explained by differences within or between substrate types altogether (β_1_: 4-6% and β_2_: 8-10%). This pattern is linked to the consistency in community structure, irrespective of the substrate type, observed in Meknès and Darwin MVs (Carbonate Province) contrasting with the higher dominance and more variable features of Mercator MV assemblages (see also k-dominance curves in Figure 4).

## Discussion

### Faunal assemblages and temporal persistence of small organic falls

For organic falls to fulfil the role of bridging dispersal over otherwise large distances, a fundamental prerequisite would be that they persist long enough to enable growth and reproduction of their inhabitants. Temporal persistence is tightly related to the amount and lability of the substrate which will also determine the rate of ecological succession and associated species turnover. More labile organic material (e.g. kelp) is scavenged by invertebrates and decomposed by microbes at much higher rates than wood falls of similar mass [16]. The occurrence of a sulphophilic phase in the organic-falls succession can last for at least 5 to 6.8 years at wood and whale falls but it is much briefer at more labile falls (e.g. 0.5 years at kelp falls) [17]. In the Gulf of Cádiz experiments, the occurrence of a sulphophilic phase was only supported by indirect evidence - darkened sediment patches beneath the devices owing to the release of particulate organic material were observed during recovery in all cases (either after 1 or 2 years). However, measurements obtained from replicate experiments in the Nile Fan cold seeps indicate that at least micromolecular concentrations of sulphide may be produced along with the decay of organic matter [22]. Moreover, a strain of sulphate reducing bacteria was isolated from the experimental wood cubes and cultivated under laboratory conditions [46].

As expected, substrate consumption was faster in alfalfa than in wood, and in shallower than in deeper sites but in most cases a large amount of the initially deployed volume of substrata remained after 1 to 2 years. The decay of organic matter constitutes a decrease in habitat suitability for organic-fall specialists therefore leading to a decline in the abundance of these species and ultimately their local extinction. The 2-year old alfalfa assemblage at Mercator MV shared many similarities with the one in the carbonate experiments which served as a control for physical availability of un-colonized surface without input of organic matter. This is in agreement with the observation that a gradual re-colonization by background species occurs when the labile substrate is consumed [17].

In general, most deep-sea organisms are deposit feeders [47], but in the deployed organic substrata we observed a high number of species including various specialist taxa and assemblages with a complex trophic structure. The available organic matter and bacterial biomass favours increased contributions of grazers putatively feeding on free-living bacteria, opportunist detritus feeders and omnivores. The high density assemblages are also able to support higher trophic levels including carnivores and scavengers. Stable isotope analyses showed that, even at relatively shallow water depth where the euphotic zone supplies abundant food, the materials derived from organic falls sustain a unique heterotrophic food web [48].

Persistence over time of the small organic falls was sufficient to allow growth, sexual maturity and reproduction in specialized and/or opportunistic taxa. Species quicker to exploit any vacant patches that become available in the environment, colonizing and reproducing before other species, may easily become dominant. In the case of Xylophagainae and lysiannasid amphipods, they also consume the substrata leading to a relatively short succession but, on the other hand, they breakdown refractory organic materials into more readily available food sources (e.g. faecal pellets) thus facilitating colonization by other species [24,49,50].

Wood-boring bivalves appear particularly well adapted for this fugitive lifestyle as demonstrated by the high settlement observed only after two weeks in wood deployed in Eastern Mediterranean [22]. High population densities, high reproductive rates, early maturity, rapid growth, and inferred planktotrophic larval development make these bivalves classic examples of species with an apparent ease of dispersal and able to utilize transient habitats [49,51,52]. Protandric hermaphroditism (also observed in *Idas modiolaeformis* (A. Hilário pers. observ.) [53], and the occurrence of dwarf males [52,54] are other reproductive traits enhancing fertilization success, and therefore potentially advantageous for species living in ephemeral habitats. In fact, the higher incidence of dwarf males in the deep-water species of *Xylophaga* may have evolved as an adaptation to the more sporadic distribution of woody substrata at greater depths [52].

The co-occurrence of adults and a high number of recently settled recruits of limpets (*Coccopigya* sp. and *Copulabyssia* sp.), bivalves (*X. dorsalis* and *I. modioliformis*) and amphipods (*O. grimaldii*) suggests successive settlement events. Both widespread dispersal (enabling high rates of habitat occupancy) and self-recruitment owing to local larval retention (enabling high local abundances and population persistence) are possible over ecologically significant time-scales in planktotrophic and lecithotrophic dispersers [55,56,57,58,59,60]. In fact, by determining dispersal probability and propagule pressure, fecundity may be the primary driver of the abundance–occupancy relationship in many marine macroinvertebrates [61]. Clearly, species with different development modes may be successful in colonizing organic falls reaching high abundances and high rates of habitat occupancy. In the Gulf of Cádiz, this is the case of cocculinid and pseudococculinid limpets which have a non-feeding larval dispersal phase [60,62], and, to a lesser extent, lysianassid amphipods which are brooders. Seemingly, Lecithotrophic development is apparently not a major impediment to relatively long-distance dispersal [55,52,57,58,59,60]. Yet, the availability and isolation of habitat patches may become more relevant for brooders, as their ability to colonize transient habitats is often limited by the foraging range of adults, particularly ovigerous females (e.g. lysianassid amphipods) [63].

### Depth and/or site related variability in small organic falls

The β-diversity patterns and diversity partitioning in the Gulf of Cádiz experiments suggests that there is a significant effect of location and presumably water depth in the colonization of organic substrata. In fact, β-diversity associated with location contributed more to the overall biodiversity than the β-diversity associated with substrate type but this result must be interpreted in the limited geographical context of our experiments. When viewed in conjunction with the data from the Mediterranean, Mid-Atlantic Ridge and Norwegian margin [22], many commonalities arise among the wood assemblages across geographical regions, while the alfalfa assemblages show higher dissimilarity and may be more strongly affected by local conditions. Moreover, the overall background context appears to provide an upper threshold for the biodiversity of the recruited assemblages. When compared to the study by Gaudron et al. [22] who reported a total of 33 taxa (for the three geographical areas), a much greater species richness (over 100 species) was recorded in the Gulf of Cádiz, a region with documented high biodiversity [35].

Depth-related changes in the abundance, biodiversity and composition of faunal assemblages from reducing environments are still poorly known [34,35] and for organic falls the available information is even more limited. A recurrent pattern in cold seeps and hydrothermal vents is the decreasing permeability to the penetration of background species, a higher degree of novelty in the fauna, and often a greater variability in the composition and structure of the assemblages with increasing depths [35,64,65]. Nonetheless, faunal assemblages in cold seeps do not seem to follow a predictable depth-related diversity trend and they are influenced by site-specific intricate effects of water depth and relevant changes in the oceanographic and geologic settings [35].

Similarly to other reducing environments, there was a higher degree of novelty (new occurrences and specialist species) in the organic substrata deployed in the deeper region (700-1000 m) than in the shallower one (350 m). However, the assemblages displayed more variability and higher rates of colonization by typical organic-fall species in the shallower than in the deeper sites the assemblages. In the shallower site, *Xylophaga dorsalis* in wood, and lysianassid amphipods in alfalfa were highly dominant (up to 90% of the total abundance) while at the deeper sites (well within their known bathymetric ranges) their colonization rates were irrelevant and the dominance was more evenly shared by several species fading away the dissimilarities between substrate types and location. Lysianassid amphipods were also absent or showed low abundances in the alfalfa experiments deployed in deep-water cold seeps (1200-2300 m) from the Nile Deep-sea Fan and the Håkon Mosby MV (Gaudron pers. com.) [22]; here again the dominant species were gastropod grazers and the assemblages showed higher evenness. Nevertheless, the greater depth of these locations did not hinder the colonization by wood-boring bivalves which reached extremely high dominance in the wood experiments.

The exclusion of lysianassid scavengers has a significant impact on ecosystem functioning by preventing the rapid recycling of organic falls [66]. Our results suggest that the settlement success of lysianassids in alfalfa as well as wood borers in wood may determine not only the rate of succession but also the coexistence of species and the resulting community structure. It is expected that, as the ecological succession progresses, species coexistence is favoured by intermediate levels of disturbance but this principle is a moderate-to-high settlement phenomenon [67]: coexistence is possible at sufficiently low settlement even without the aid of disturbance, and at high settlement, a dominant with greater benthic ability may exclude the subordinate at all levels of disturbance. The differences observed in the structure of the assemblages may be thus interpreted as resulting from the intensity of settlement, and ultimately propagule pressure, of organic-fall specialists at different depths or locations. If a widespread (although patchy) occurrence of coastal-derived wood and other phytodetritus is hypothesized, than propagule pressure of their typical inhabitants may be a function of distance from the shore, more than a function of depth. This seems to hold true for the results obtained by Gaudron et al. [22] who reported colonization rates after one year in the following sequence Mid-Atlantic Ridge < Nile Deep-sea Fan < Håkon Mosby MV. However the relationship between propagule pressure and distance from shore is a simplistic assumption as it underestimates the effects of local and regional oceanography on dispersal. The processes determining propagule availability and settlement are a far more complex interplay of abiotic drivers and biotic interactions, but irrespective of the causes for the low settlement of wood-boring bivalves and lysianassid amphipods, the absence of dominant substrate-consuming species allowed longer persistence, and apparently led to more even assemblages. These were still dominated by typical organic-fall species, but with higher relative contributions of the local background fauna.

### The influence of a cold seepage background

The occurrence of a sulphophilic phase in the organic-fall succession is essential for the occurrence of chemoautotrophy and it is the central stage for their resemblance to other deep-sea reducing environments [17]. The experiments with small amounts of organic material deployed in the Gulf of Cádiz, and in other regions with reducing environments [22], were capable of sustaining populations of the chemosymbiotic bivalve *Idas modiolaeformis* (referred by Gaudron et al. [22] as *Idas* sp. Med) in which symbiont acquisition [45] and reproductive maturity [53] were confirmed. *Idas modiolaeformis* was also the only chemosymbiotic metazoan associated with experimental large wood falls deployed at different distances from the Nile Deep-sea Fan cold seeps [26]. Species of the genus *Idas* are considered as typical organic-fall inhabitants [68,69] although *I. modiolaeformis* is also known to occur attached to biogenic and carbonate substrata in the Mediterranean cold seeps [70]. The settlement of other chemosymbiotic or mixotrophic bivalves such as Solemyidae in the Gulf of Cádiz (this study), and Vesicomyidae and Thyasiridae in the Nile Deep-sea Fan and Eastern Mediterranean [22] was observed, but only a few very small individuals were recovered from the organic substrata. Apparently, none survived the period of high mortality immediately after settlement, and adults were never found. The settlement of these bivalves in the experiments, likely to be sourced by the local mud volcano populations, may be triggered by the sulphidic conditions developing in the experiments. However, a putative effect of a chemical cue from the experimental organic substrata is not supported by the settlement pattern of *Sclerolinum contortum* in the experiments deployed in the Håkon Mosby MV (Norwegian margin) [22] where this chemosymbiotic tubeworm recruited both in organic and inorganic substrata. Vestimentiferans, with thiotrophic symbionts, have been also recorded occasionally in a shipwreck with an organic cargo [71] and in sediments containing whalebones, although never ecologically dominant [72]. These results suggest that chemosymbiotic species strongly relying on seepage from a sedimentary environment will not be able to thrive in association with small organic falls.

More than the low concentrations of sulphide which may be produced by the deployed organic substrata, it is the natural occurrence of sulphide in the surrounding setting of the experiments that explains the settlement of tubeworms in Håkon Mosby MV. Sulphide concentrations at different experimental locations may also provide an explanation for differences in lysianassid colonization as they were only dominant in the alfalfa assemblages at Mercator MV where the seepage is mild [35]. Although lysianassids can tolerate reduced environments, they require daily migrations to better oxygenated waters for recovery of routine metabolic rates and removal of sulphide and acidic end-products of anaerobic metabolism [73]. The 2 mm mesh net enclosing the experimental substrata is an obstacle for such migrations and would probably affect their survival under higher local sulphide concentrations.

The capability of animals to live in the presence of sulphide has been proven in many aquatic species with well-developed detoxification metabolism [74] although evidence for deep-sea species remains scarce. At the Eel river methane seeps, dorvilleid polychaetes (*Ophryotrocha* spp.) thrived at sulphide concentration over 10 mM but most infaunal taxa avoided concentrations over 1 mM [75]. Some trophic groups such as bacterial grazers and deposit-feeders or omnivores that rely on bacterial biomass for their nutrition are likely to be exposed to higher sulphide concentrations and may develop physiological or behavioural adaptations. In what concerns heterotrophic species it is clear that sulphide tolerance is an advantage for colonizing reducing environments [17] and a favourable foundation for the putative similarity of their faunas. Almost all the deposit feeders found in our experiments were also known from the mud volcano sediments but, on the other hand, omnivores (including *Ophryotrocha* species) and bacterial grazers were two of the trophic groups with the highest percentage of new occurrences. In fact, typical organic-fall taxa were always dominant in the assemblages of the experimental organic substrata despite the surrounding cold seep setting. The non-experimental study of a whale fall in the Monterey Canyon [76] provides alternative evidence for limited influence (if any) of seep proximity. In this whale fall the assemblage shared no species with the nearby cold-seeps located only 300 m away.

## Conclusion

Small organic falls are probably ubiquitous and therefore the distance between colonizable transient patches of reducing conditions may be short (100s to 1000s m), and reachable by taxa with relatively low dispersal capabilities. Small volumes of organic material can sustain assemblages that are trophically coherent for time periods that allow growth to reproductive maturity, and potential succession of one or more generations of the dominant species. For that reason, they may act as stepping stones for fugitive species, mainly organic-fall specialists and opportunists. They may also represent islands of food resources contributing to increase locally the population densities of many deep-sea background species.

The composition and structure of the organic-fall assemblages rely on the regional and local availability of propagules and hence they show variability consistent with their biogeographic and bathymetric contexts. However, the proximity of cold seeps has limited influence on the recruitment of organic falls, and does not lead to a meaningful increase of the similarity between the assemblages of these two habitats. Despite the phylogenetic and trophic commonalities of different reducing environments, organic falls sustain a distinctive fauna which includes dominant substrate-specific taxa (from phytodetritus, wood, animal carcasses). There may be a critical size of organic falls necessary to create sufficiently intense and persistent reducing conditions to sustain typical vent and seep chemosymbiotic fauna; small organic falls below this critical size, may be therefore unable to ensure population connectivity of such taxa.

In patchy environments, such as organic falls, where food sources are diverse and habitat patches differ in their productivity, adaptive mechanisms leading to ecological polymorphism or niche differentiation may be favoured by rapid population growth and generation succession. Non-adaptive allopatric mechanisms may further include the founder effect (small isolates create starting gene pools for incipient species) and genetic drift of small populations, but sympatric hybridization should not be discarded when incipient species come together in a habitat patch. Evidence on multiple invasions of cold seeps and radiation via specialization and resource partitioning is provided for the genus *Ophryotrocha* [77,78]. This sulphide-tolerant polychaete is often represented by several sympatric species either in shallow reducing environments, deep-water seeps, or in different types of organic falls [14,77,79,80]. Bathymodiolin mussels [68], *Osedax* boneworms [81,82] and Xylophagainae wood borers [52,83,84,85] are other examples of organic-fall inhabitants with a remarkable number of species but only the mussels are represented in other reducing environments.

The widespread patchy distribution of organic falls and the transient nature of their fauna are a fertile ground for speciation involving both adaptive and non-adaptive mechanisms in a relatively short evolutionary time. Irrespective of the debate on the origin and chronology of the modern fauna of reducing environments, organic falls may be a recurrent source of evolutionary candidates for the colonization of these deep-sea habitats. Although the role of organic falls in currently bridging dispersal between different types of chemoautotrophy-based assemblages remains controversial, their part in the adaptation and evolution of deep-sea fauna is indisputable.

## Supporting Information

Text S1 **Environmental and biological characterization of the three study sites.**
(DOCX)

Figure S1 **Location of the three study sites (Mercator, Meknès and Darwin mud volcanoes) in the Gulf of Cádiz.** Black dots show the position of other mud volcanoes in the region.
(TIF)

Table S1 **List of the taxa identified from the colonization experiments (CHEMECOLI) deployed in the Gulf of Cádiz.** Classification according to the World Register of Marine Species (www.marinespecies.org accessed May 2013). The taxa in blue were found only in the external parts of the CHEMECOLI. The occurrence of new records (in bold) and background fauna in the substrata enclosed by the 2mm mesh net is shown for each sub-region (El Arraiche, Carbonate Province) and substrate type (wood, alfalfa, carbonate). Each taxon was assigned to one of 20 different trophic groups.
(DOCX)

Table S2 **Breakdown of percentual contributions from SIMPER analysis for comparisons between mud volcanoes: Mercator (Mer); Meknès (Mek) and Darwin (Dar).** The taxa listed contribute at least 1.5%. Numbers in bold mark the six dominant species at each site.
(DOC)

Table S3 **Breakdown of percentual contributions from SIMPER analysis for comparisons between substrate types: wood (W), alfalfa (A) and carbonate (C).** The taxa listed contribute at least 1.5%. Numbers in bold mark the six dominant species in each substrate type.
(DOC)
