# Supplementary material for: Are Organic Falls Bridging Reduced Environments in the Deep Sea? - Results from Colonization Experiments in the Gulf of Cádiz
Source: PLoS One. 2013 Oct 2;8(10):e76688. doi: 10.1371/journal.pone.0076688 (PMC3788751; doi:10.1371/journal.pone.0076688)
Supplement: Text S1 — Environmental and biological characterization of the three study sites. (DOCX) [file pone.0076688.s001.docx]

**Supporting Information**

**Text S1. Environmental and biological characterization of the three study sites**

**Mercator MV.** The shallowest study site, Mercator (350 m), is located at the El Arraiche field on the upper slope of the Moroccan margin [1] where the proximity to the euphotic zone and to the African coast adds to the great productivity observed in the area. Colonisation experiments were deployed in the crater at the top of Mercator where the seafloor shows patches of disturbed sediments from which gas venting is occasionally observed [2]. The sediment consists of brown pelagic silty clay covering grey matrix-supported breccia with clasts of different lithology and size with disseminated sulphides (pyrite-marcasite) and filled fractures. The sulphides form framboidal aggregates, generally less than 2mm and several carbonated cemented clasts within clayey matrix. Mercator MV is an example of gas production by admixture of thermogenic sources from different depths and influence of evaporite deposits. Nuzzo et al. [3] the porewater is extremely enriched in chloride (Cl^-^ reaching up to 5.3 M thus, exceeding normal seawater values by a factor of 9) and SO_4_ consistent with the dissolution of minerals (e.g. halite and gypsum), additionally, the fluids are highly enriched in Li and B indicating a deep fluid source from mineral dewatering reactions at elevated temperatures [4]; radiogenic ^87^Sr/^86^Sr ratios are consistent with a terrigenous/continental deep source of the fluids [5], and the carbon isotopic signature of methane (δ^13^C: -33.8 to -38.8‰) indicates high thermal maturity [3,6,7]. The upward fluid flow rates were estimated as ~6 cm/a at the top of the MV, gradually decreasing to 0.3 cm/a towards the rim [4].

The megafauna at Mercator mud volcano is sparse, with some fish (*Helicolenus* sp.), crinoids and cidarid echinoids associated mainly to the boulders in the crater and the sea-pen *Pennatula aculeata* anchored in the sediments at the rim. Solitary corals (*Caryophyllia* sp.) accompanied by Cidaridae echinoids and Onuphidae polychaetes (*Hyalinoecia tubicola*) are the most conspicuous organisms seen during video surveys of the crater. Over 300 macrofaunal species are known to occur in Mercator MV [8, MR Cunha unpublished data]. The chemosymbiotic fauna recorded in this mud volcano includes the bivalves *Solemya elarraichensis,* *Lucinoma asapheus,* *Axinulus croulinensis* (mixotrophic species); *Thyasira granulosa* (mixotrophic species) and five Frenulata species, *Polybrachia* sp.1, *Siboglinum* Ib, *Siboglinum* Ic, *Siboglinum* Id, and *Siboglinum* sp.1 [9]*.*

**Meknés MV.** In the Moroccan margin the extensive authigenic Carbonate Province at intermediate depths (700-1200m) is accompanied by the frequent occurrence of mounds, thickets and debris of mostly dead cold-water scleractinean corals. Meknès MV is the southernmost mud volcano in this region rising isolated among an extensive field of small coral mounds at ca. 700m depth. The porewater is characterized by a moderate depletion of Cl^-^ [5,7]. The carbon isotopic signature of methane is more depleted (δ^13^C: -48.9 to -52.8‰) than in Mercator MV suggesting lower thermal maturity [3].

Colonisation experiments were deployed at the crater which is formed by stiff, sometimes heavily disturbed, greenish grey mud breccia with scattered clasts of different lithology and size (2mm -5 cm in diameter) and a striking large number of empty shells of the gastropod *Neptunea contraria*. The surveys over Meknès showed coral rubble and small coral thickets colonised by sponges and octocorals at the lower flanks of the mud volcano contrasting with the almost bare mud breccia of the crater inhabited only by *N. contraria*, *Paromola cuvieri* and *Helicolenus* sp. Except for a few individuals of these three species, living megafauna is rarely sighted in the crater. Over 175 macrofaunal species are known to occur in Meknès MV [8, M.R. Cunha unpublished data]. The chemosymbiotic fauna recorded in this mud volcano includes the bivalve *Solemya elarraichensis* and two Frenulata species *Siboglinum* If and *Siboglinum* sp.2 [9].

**Darwin MV.** The carbonate province also includes the Darwin mud volcano (ca. 1100m). Darwin MV differs from the other mud volcanoes in this area because its crater is completely covered by large carbonate slabs and crusts; the fissures among slabs and depressions with scattered crust are filled with abundant shell ash and occasionally small clumps of living *“Bathymodiolus” mauritanicus*. *Paromola cuvieri*, soft corals and other epifauna were occasionally sighted on the surface of rocks and sediment. Over 100 macrofaunal species are known to occur in Darwin MV (MR. Cunha unpublished data) where the gastropod fauna is particularly diverse [10]. The chemosymbiotic fauna recorded in this mud volcano includes the bivalves *Isorropodon megadesmus,* *Solemya elarraichensis* and “*Bathymodiolus”mauritanicus*, and two Frenulata species, *Siboglinum* Ia and *Siboglinum* Ie [9].

**References**

1. Van Rensbergen P, Depreiter D, Pannemans B, Moerkerke G, Van Rooij D, Marsset B, Akhmanov G, Blinova V, Ivanov M, Rachidi M, Magalhães V, Pinheiro L, Cunha M, Henriet J-P (2005) The El Arraiche mud volcano field at the Moroccan Atlantic slope, Gulf of Cadiz. Mar Geol 219: 1-17.

2. Vanreusel A, Andersen AC, Boetius A, Connelly D, Cunha MR, Decker C, Hilário A, Kormas KA, Maignien L, Olu K, Pachiadaki M, Ritt B, Rodrigues C, Sarrazin J, Tyler P, Van Gaever S, Vanneste H (2009) Biodiversity of cold seep ecosystems along the European margins. Oceanography 22: 110-127.

3. Nuzzo M, Hornibrook ERC, Gill F, Hensen C, Pancost RD, Haeckel M, Reitz A, Scholz F, Magalhães VH, Brueckmann W, Pinheiro LM (2009) Origin of light volatile hydrocarbon gases in mud volcano fluids, Gulf of Cadiz - Evidence for multiple sources and transport mechanisms in active sedimentary wedges. Chem Geol 266: 359-372.

4. Haffert L, Haeckel M, Liebetrau V, Berndt C, Hensen C, Nuzzo M, Reitz A, Scholz F, Schönfeld J, Perez-Garcia C, Weise SM (2013) Fluid evolution and authigenic mineral paragenesis related to salt diapirism – The Mercator mud volcano in the Gulf of Cadiz. [Geochimica et Cosmochimica Acta](http://www.sciencedirect.com/science/journal/00167037) 106: 261-286.

5. Scholz F, Hensen C, Lu Z, Fehn U (2010) Controls on the ^129^I/I ratio of deep-seated marine interstitial fluids: ‘Old’ organic versus fissiogenic 129-iodine. Earth Planet Sc Lett 294: 27-36.

6. Stadnitskaia A, Ivanov MK, Blinova V, Kreulen R, van Weering TCE (2006) Molecular and carbon isotopic variability of hydrocarbon gases from mud volcanoes in the Gulf of Cadiz, NE Atlantic. Mar Petrol Geol 23: 281–296.

7. Hensen C, Nuzzo M, Hornibrook E, Pinheiro LM, Bock B, Magalhães VH, Bruckmann W (2007) Sources of mud volcano fluids in the Gulf of Cadiz – indications for hydrothermal imprint. Geochim Cosmochim Acta 71: 1232– 1248.

8. Cunha MR, Rodrigues CF, Génio L, Hilário A, Ravara A, Pfannkuche O (2013) Macrofaunal assemblages from mud volcanoes in the Gulf of Cadiz: abundance, biodiversity and diversity partitioning across spatial scales. Biogeosciences 10: 2553–2568. doi:10.5194/bg-10-2553-2013.

9. Rodrigues CF, Hilário A, Cunha MR (2013) Chemosymbiotic species from the Gulf of Cadiz (NE Atlantic): distribution, life styles and nutritional patterns. Biogeosciences, 10, 2569-2581.doi: 10.5194/bg-10-2569-2013

10. Génio L, Warén A, Matos FL, Cunha MR (2013) A snails’ tale at deep-sea habitats in the Gulf of Cadiz (NE Atlantic). Biogeosciences Discuss 10: 3707-3733.
