# Supplementary figures and images for: Are Organic Falls Bridging Reduced Environments in the Deep Sea? - Results from Colonization Experiments in the Gulf of Cádiz
Source: PLoS One. 2013 Oct 2;8(10):e76688. doi: 10.1371/journal.pone.0076688 (PMC3788751; doi:10.1371/journal.pone.0076688)

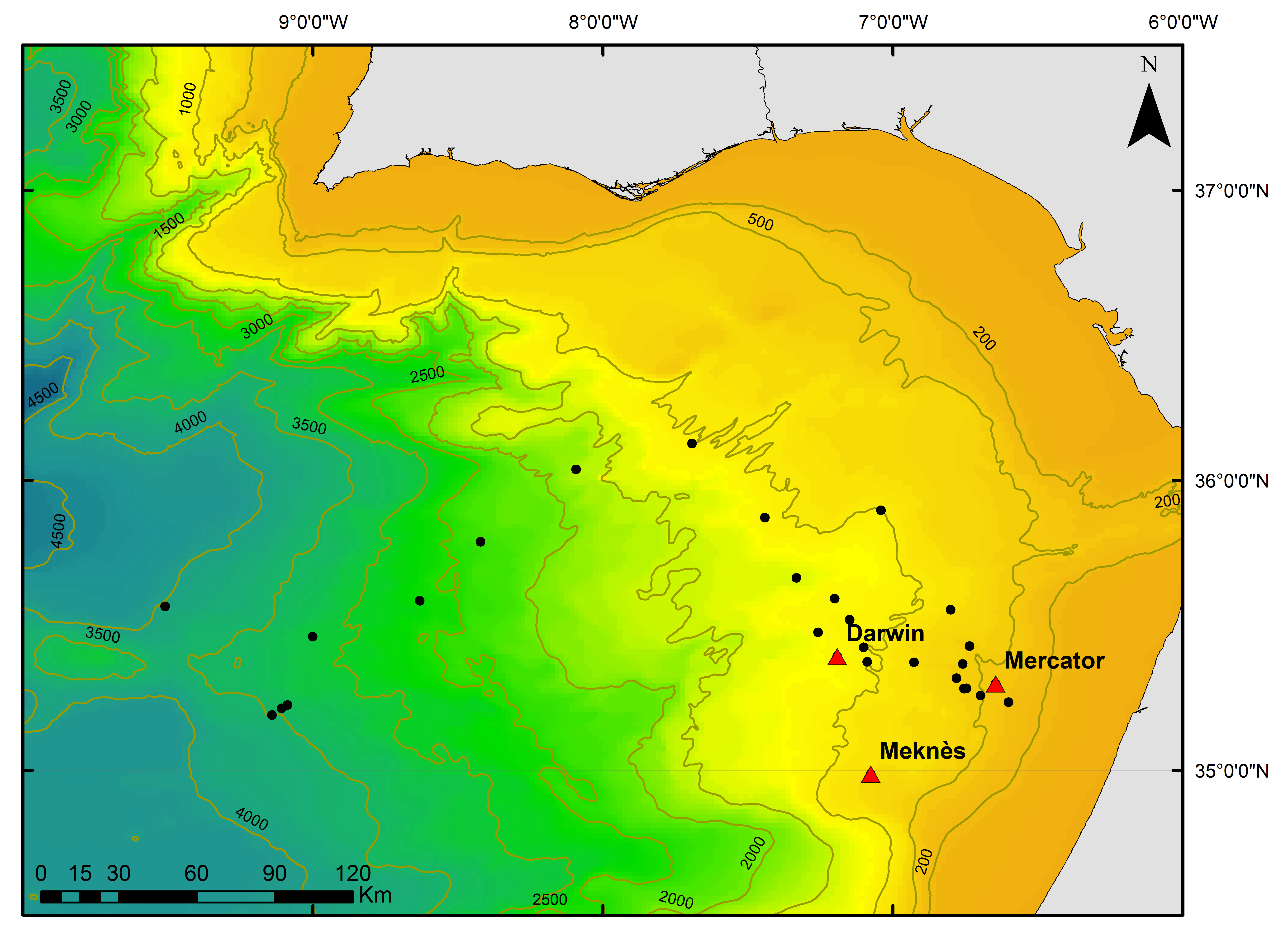

Supplement: Figure S1 — Location of the three study sites (Mercator, Meknès and Darwin mud volcanoes) in the Gulf of Cádiz. Black dots show the position of other mud volcanoes in the region. (TIF) [file pone.0076688.s002.tif]
