# Supplementary material for: Are Organic Falls Bridging Reduced Environments in the Deep Sea? - Results from Colonization Experiments in the Gulf of Cádiz
Source: PLoS One. 2013 Oct 2;8(10):e76688. doi: 10.1371/journal.pone.0076688 (PMC3788751; doi:10.1371/journal.pone.0076688)
Supplement: Table S1 — List of the taxa identified from the colonization experiments (CHEMECOLI) deployed in the Gulf of Cádiz. Classification according to the World Register of Marine Species (www.marinespecies.org accessed May 2013). The taxa in blue were found only in the external parts of the CHEMECOLI. The occurrence of new records (in bold) and background fauna in the substrata enclosed by the 2mm mesh net is shown for each sub-region (El Arraiche, Carbonate Province) and substrate type (wood, alfalfa, carbonate). Each taxon was assigned to one of 20 different trophic groups. (DOCX) [file pone.0076688.s003.docx]

**Table S1**. List of the taxa identified from the colonization experiments (CHEMECOLI) deployed in the Gulf of Cadiz. Classification according to the World Register of Marine Species ([www.marinespecies.org](http://www.marinespecies.org) accessed May 2013). The taxa in blue were found only in the external parts of the CHEMECOLI. The occurrence of new records (in bold) and background fauna in the substrata enclosed by the 2mm mesh net is shown for each sub-region (El Arraiche, Carbonate Province) and substrate type (wood, alfalfa, carbonate). Each taxon was assigned to one of 20 different trophic groups.

|  |  |  | **Feeding** | | | | **EA** | | | **CP** | | |
| --- | --- | --- | --- | --- | --- | --- | --- | --- | --- | --- | --- | --- |
| **Major taxa** | **Family** | **Species name/code** | **Habit** | **Mode** | **Food type** | **TG** | **W** | **A** | **C** | **W** | **A** | **C** |
| **PORIFERA** |  |  |  |  |  |  |  |  |  |  |  |  |
| Unassigned | Unassigned | Porifera unassigned | Ep/S/R | Om/Su | pom | 16 | - | - | - | - | - | U |
| **CNIDARIA** |  |  |  |  |  |  |  |  |  |  |  |  |
| **Hydrozoa** |  |  |  |  |  |  |  |  |  |  |  |  |
| Unassigned | Unassigned | Hydrozoa unassigned | Ep/S/A | Ca/Su | zoo | 5 | U | U | U | - | - | U |
| Anthoathecata | Tubiclavoididae | *Tubiclavoides striatum* Moura, Cunha & Schuchert, 2007 | Ep/S/A | Ca/Su | zoo | 5 | - | - | - | - | - | - |
|  | Eudendriidae | *Eudendrium* sp*.* | Ep/S/A | Ca/Su | zoo | 5 | - | - | - | - | - | L |
| Leptothecata | Campanulariidae | *Clytia* sp. | Ep/S/A | Ca/Su | zoo | 5 | R | - | R | - | - | - |
|  |  | *Clytia linearis* (Thorneley, 1900) | Ep/S/A | Ca/Su | zoo | 5 | R | R | R | - | - | R |
|  | Campanulinidae | Campanulinidae unassigned | Ep/S/A | Ca/Su | zoo | 5 | - | - | - | U | - | - |
|  |  | *Campanulina panícula* G.O. Sars, 1874 | Ep/S/A | Ca/Su | zoo | 5 | - | L | - | - | - | - |
|  | Halecidae | *Halecium* cf. *tenellum* Hincks, 1861 | Ep/S/A | Ca/Su | zoo | 5 | R | - | - | - | - | - |
|  | Lafoeidae | *Cryptolaria pectinata* (Allman, 1888) | Ep/S/A | Ca/Su | zoo | 5 | - | R | - | - | - | - |
|  |  | *Filellum serratum* (Clarke, 1879) | Ep/S/A | Ca/Su | zoo | 5 | L | L | - | - | - | - |
|  |  | *Zygophylax biarmata* Billard, 1905 | Ep/S/A | Ca/Su | zoo | 5 | - | L | - | - | - | R |
|  | Tiarannidae | *Modeeria rotunda* (Quoy & Gaimard,1827) | Ep/S/A | Ca/Su | zoo | 5 | - | - | - | - | - | - |
| **Anthozoa** |  |  |  |  |  |  |  |  |  |  |  |  |
| Actiniaria | Unassigned | Actiniaria unassigned | Ep/S/A | Ca/Su | zoo | 5 | - | - | - | U | - | - |
| **Scyphozoa** |  |  |  |  |  |  |  |  |  |  |  |  |
| Coronata | Nausithoidae | *Nausithoe* sp. | Ep/S/A | U |  | U |  |  |  |  |  |  |
| **NEMERTEA** |  |  |  |  |  |  |  |  |  |  |  |  |
| Unassigned | Unassigned | Nemertea unassigned | Ss/M/F | Ca/Pr | mac | 6 | U | - | U | - | - | - |
| **SIPUNCULA** |  |  |  |  |  |  |  |  |  |  |  |  |
| Unassigned | Unassigned | Sipuncula unassigned | Sr/D/F | Om/Dt | pom;mic;mac | 12 | U | - | - | U | - | U |
| **ANNELIDA** |  |  |  |  |  |  |  |  |  |  |  |  |
| **Oligochaeta** |  |  |  |  |  |  |  |  |  |  |  |  |
| Unassigned | Unassigned | Oligochaeta unassigned | Ss/M/F | Om/Dt | pom;mic | 1 |  |  |  |  |  |  |
| **Polychaeta** |  |  |  |  |  |  |  |  |  |  |  |  |
| Incerate sedis | [Protodrilidae](http://www.marinespecies.org/aphia.php?p=taxdetails&id=994) | ***Protodrilus* sp.** | Sr/M/F | Mc/Gr | mic | 11 | - | - | - | **N** | **N** | - |
| Aciculata | Amphinomidae | ***Linopherus* cf. *hemuli* (Fauchald, 1972)** | Ss/M/F | Ca/Pr | mac | 6 | - | - | - | **N** | **-** | - |
|  |  | *Pareurythoe* cf. *borealis* (M.Sars,1862) | Ss/M/F | Ca/Pr | mac | 6 | R | - | - | R | - | - |
|  | Dorvilleidae | ***Ophryotrocha* sp01** | Sr/M/F | Om/Sc;Gr | pom;mic;mei | 2 | **N** | **N** | - | **N** | - | - |
|  |  | ***Ophryotrocha* sp02** | Sr/M/F | Om/Sc;Gr | pom;mic;mei | 2 | **N** | **N** | - | **N** | **N** | - |
|  |  | ***Ophryotrocha* sp03** | Sr/M/F | Om/Sc;Gr | pom;mic;mei | 2 | **N** | - | - | - | - | - |
|  |  | ***Ophryotrocha* sp04** | Sr/M/F | Om/Sc;Gr | pom;mic;mei | 2 | **N** | - | - | - | - | - |
|  |  | ***Ophryotrocha* sp05** | Sr/M/F | Om/Sc;Gr | pom;mic;mei | 2 | **N** | - | - | **N** | **N** | - |
|  |  | ***Ophryotrocha* sp06** | Sr/M/F | Om/Sc;Gr | pom;mic;mei | 2 | - | - | - | **N** | **N** | - |
|  |  | ***Ophryotrocha* sp07** | Sr/M/F | Om/Sc;Gr | pom;mic;mei | 2 | - | - | - | - | - | - |
|  |  | ***Ophryotrocha* sp08** | Sr/M/F | Om/Sc;Gr | pom;mic;mei | 2 | - | - | - | **N** | **N** | - |
|  |  | ***Protodorvillea kefersteini* (McIntosh, 1869)** | Ss/M/F | Ca/Pr | mei | 8 | - | - | **N** | - | - | - |
|  | Lumbrineridae | Lumbrineridae unassigned | Ss/M/F | Ca/Pr | mei;mac | 8 | - | - | - | - | U | - |
|  |  | *Lumbrineriopsis paradoxa* (Saint-Joseph,1888) | Ss/M/F | Ca/Pr | mei;mac | 8 | - | - | - | - | **N** | - |
|  | Chrysopetalidae | Chrysopetalidae unassigned | Sr/M/F | Ca/Pr | mei;mac | 9 | U | - | - | - | - | - |
|  | Glyceridae | Glyceridae unassigned | Ss/M/F | Ca/Pr | mac | 6 | - | - | - | - | - | - |
|  |  | *Glycera tesselata* Grübe, 1840 | Ss/M/F | Ca/Pr | mac | 6 | - | - | - | L | - | - |
|  | Hesionidae | Hesionidae unassigned | Ss/M/F | Ca/Pr | mac | 6 | U | - | - | U | - | - |
|  |  | **cf. *Amphiduros* sp.** | Sr/M/F | Ca/Pr;Sc | mac | 4 | **N** | **N** | - | **N** | **N** | - |
|  |  | *Leocrates atlanticus* (McIntosh, 1885) | Ss/M/F | Ca/Pr | mac | 6 | L | - | - | L | L | L |
|  |  | ***Nereimyra* sp**.(juveniles) | Ss/M/F | Ca/Pr | mac | 6 | **N** | - | - | **N** | **N** | - |
|  |  | ***Nereimyra punctata* (Muller, 1788)** | Ss/M/F | Ca/Pr | mac | 6 | - | - | - | - | - | - |
|  | Nereididae | *Eunereis longíssima* Johnston, 1840 | Ss/D/T | Om/De | sed;pom;mic | 13 | L | - | - | - | - | - |
|  |  | *Nicon sínica* Wu & Sun, 1979 | Sr/D/T | Om/Dt;Pr | pom;mic;mei | 2 | - | - | - | - | - | - |
|  | Phyllodocidae | *Phyllodoce madeirensis* Langerhans, 1880 | Sr/M/F | Ca/Pr;Sc | mac | 4 | R | - | - | - | - | - |
|  | Polynoidae | *Harmothoe evei* Kirkegaard, 1980 | Sr/M/F | Ca/Pr | mac | 7 | R | R | R | R | - | - |
|  |  | *Subadyte pellucida* (Ehlers, 1864) | Ss/M/F | Ca/Pr | mac | 6 | L | L | L | R | R | - |
|  | Sigalionidae | *Pholoides dorsipapillatus* (Marenzeller, 1893) | Ss/M/F | Ca/Pr | mac | 6 | - | - | - | - | - | - |
|  |  | *Sthenelais* cf. *boa* (Johnston,1833) | Ss/M/F | Ca/Pr | mac | 6 | - | - | - | - | - | - |
|  | Syllidae | Eusyllinae unassigned | Ss/M/F | Ca/Pr | mei | 8 | U | - | - | U | - | - |
|  |  | Exogoninae unassigned | Sr/M/F | Mc/Gr | mic | 10 | U | - | - | U | U | - |
| Canalipalpata | Sabellidae | Sabellidae unassigned | Ep/S/T | Om/Su | pom | 16 | - | - | U | - | - | - |
|  | Sabellidae | Serpulidae unassigned | Ep/S/T | Om/Su | pom | 16 | U | - | - | - | - | - |
|  | Siboglinidae | *Siboglinum* sp. | Ss/S/T | Ch | - | 20 | - | - | - | - | R | - |
|  | Spionidae | *Polydora* sp. | Sr/D/T | Om/De;Su | sed;pom;mic | 14 | - | - | - | - | - | - |
|  |  | *Prionospio* sp01 | Sr/D/T | Om/De;Su | sed;pom;mic | 14 | - | U | - | U | - | - |
|  |  | *Prionospio* sp02 | Sr/D/T | Om/De;Su | sed;pom;mic | 14 | - | - | - | - | - | - |
|  |  | *Prionospio* cf. *aluta* Maciolek, 1985 | Sr/D/T | Om/De;Su | sed;pom;mic | 14 | L | - | - | L | - | - |
|  |  | *Prionospio steenstrupi* Malmgren,1867 | Sr/D/T | Om/De;Su | sed;pom;mic | 14 | - | - | - | - | - | - |
|  | Acrocirridae | *Macrochaeta* sp. | Sr/M/F | Om/De | sed;pom;mic | 14 | - | - | - | - | - | - |
|  | Ampharetidae | *Amage* sp. | Sr/D/T | Om/De | sed;pom;mic | 14 | L | - | - | R | - | R |
|  |  | *Melinnopsis* sp. | Sr/D/T | Om/De | sed;pom;mic | 14 | L | L | - | R | R | - |
|  | Ctenodrilidae | ***Raricirrus beryli* Petersen & George, 1991** | Sr/M/F | Mc/Gr | mic | 10 | **N** | - | - | **N** | **N** | - |
|  | Terebellidae | Terebellidae unassigned | Sr/D/T | Om/De | sed;pom;mic | 14 | U | - | U | - | U | - |
|  |  | cf. *Amphitrite* sp. | Sr/D/T | Om/De | sed;pom;mic | 14 | - | - | - | - | - | - |
|  |  | *Neoamphitrite affinis* (Malmgren,1866) | Sr/D/T | Om/De | sed;pom;mic | 14 | R | - | - | - | - | - |
|  |  | ***Nicolea* cf. *venustula* (Montagu, 1818)** | Sr/D/T | Om/De | sed;pom;mic | 14 | - | - | - | - | - | **N** |
|  |  | *Polycirrus norvegicus* Wollebaek, 1912 | Sr/D/T | Om/De | sed;pom;mic | 14 | L | L | L | - | - | - |
| Scolecida | Capitellidae | Capitellidae sp01 | Ss/D/F | Om/De | sed;pom;mic | 13 | U | - | U | U | U | - |
|  |  | Capitellidae sp02 | Ss/D/F | Om/De | sed;pom;mic | 13 | U | U | - | - | - | - |
|  |  | Capitellidae sp03 | Ss/D/F | Om/De | sed;pom;mic | 13 | U | - | - | U | - | - |
|  |  | Capitellidae sp04 | Ss/D/F | Om/De | sed;pom;mic | 13 | U | - | - | - | - | - |
|  |  | Capitellidae sp05 | Ss/D/F | Om/De | sed;pom;mic | 13 | - | U | U | - | - | - |
|  | Maldanidae | Maldanidae unassigned | Ss/D/T | Om/De | sed;pom;mic | 13 | U | - | - | - | - | - |
|  |  | *Euclymene* cf. *oerstedi* (Claparède, 1863) | Ss/D/T | Om/De | sed;pom;mic | 13 | - | - | - | - | - | - |
|  | Orbiniidae | Orbiniidae unasssigned | Ss/M/F | Om/De | sed;pom;mic | 13 | - | - | - | - | - | - |
|  |  | *Leitoscoloplos* cf. *mammosus* Mackie, 1987 | Ss/M/F | Om/De | sed;pom;mic | 13 | - | - | L | - | - | - |
|  | Paraonidae | *Aricidea suecica meridionalis* Laubier & Ramos,1974 | Sr/D/B | Om/De | sed;pom;mic | 14 | L | - | - | - | - | - |
|  |  | *Levinsenia gracilis* (Tauber, 1879) | Sr/D/B | Om/De | sed;pom;mic | 14 | - | - | - | - | - | - |
|  |  | *Paradoneis lyra* (Southern, 1914) | Sr/D/B | Om/De | sed;pom;mic | 14 | - | - | L | - | - | - |
|  |  | Scalibregmatidae unassigned | Ss/M/B | Om/De | sed;pom;mic | 13 | - | - | - | - | - | - |
| **MOLLUSCA** |  |  |  |  |  |  |  |  |  |  |  |  |
| **Gastropoda** |  |  |  |  |  |  |  |  |  |  |  |  |
| Caenogastropoda | Eulimidae | Eulimidae unassigned | Sr/M/X | Ca/Sp | mac | 17 | - | - | - | - | R | R |
|  | Rissoidae | Rissoidae unassigned | Sr/M/F | Mc/Gr | mic | 10 | U | - | - | - | - | - |
|  |  | cf. *Alvania* sp. | Sr/M/F | Mc/Gr | mic | 10 | - | - | - | - | - | - |
|  |  | *Alvania cimicoides* (Forbes, 1844) | Sr/M/F | Mc/Gr | mic | 10 | - | - | - | - | - | - |
|  |  | *Alvania* cf. *zylensis* Gofas & Warén, 1982 | Sr/M/F | Mc/Gr | mic | 10 | R | - | - | - | - | - |
|  |  | cf. *Obtusella* sp. | Sr/M/F | Mc/Gr | mic | 10 | - | - | - | - | R | - |
|  |  | ***Pseudosetia* spD** | Sr/M/F | Mc/Gr | mic | 10 | **N** | **N** | - | - | - | - |
|  | Columbellidae | *Amphissa acutecostata* (Philippi, 1844) | Sr/M/F | Ca/Pr | mac | 7 | - | - | - | - | - | - |
|  | Marginellidae | Marginellidae spA | Sr/M/F | Ca/Pr | mac | 7 | - | L | - | - | - | - |
|  | Muricidae | ***Pagodula echinata* (Kiener, 1840)** | Sr/M/F | Ca/Pr | mac | 7 | - | - | - | - | - | - |
| Cocculiniformia | Cocculinidae | ***Coccopigya* sp.** | Sr/M/F | Mc/Gr | mic | 11 | **N** | - | - | **N** | **N** | - |
| Heterobranchia | Unassigned | Nudibranchia unassigned | Sr/M/F | Ca/Pr | mei;mac | 7 | - | - | - | - | - | U |
|  | Cimidae | *Cima cuteculata* Warén, 1993 | Sr/M/F | Mc/Gr | mic | 11 | - | - | - | - | - | - |
|  |  | *Graphis gracilis* (Monterosato, 1874) | Sr/M/F | U |  | U | - | - | - | - | L | - |
|  | Xylodisculidae | ***Xylodiscula* sp.** | Sr/M/F | Mc/Gr | mic | 11 | **N** | **N** | - | **N** | **N** | - |
| Neomphalina | Melanodrymiidae | **cf. *Leptogyra* sp.** | Sr/M/F | Mc/Gr | mic | 10 | - | - | - | **N** | **N** | - |
| Vetigastropoda | Pseudococculinidae | ***Copulabyssia* sp** | Sr/M/F | Mc/Gr | mic | 11 | **N** | **N** | - | **N** | **N** | - |
|  | Larocheidae | ***Bathyxylophila* sp. nov** | Sr/M/F | Mc/Gr | mic | 11 | - | - | - | **N** | **N** | - |
|  | Unassigned | *Moelleriopsis messanensis* (Seguenza,1876) | Sr/M/F | Mc/Gr | mic | 10 | - | - | - | - | - | - |
|  | Calliotropidae | *Putzeysia* cf. *wiseri* (Clacara, 1842) | Sr/M/F | Mc/Gr | mic | 10 | - | - | - | - | - | L |
|  | Unassigned | Vetigastropoda (skeneimorph) | Sr/M/F | U |  | U | - | - | - | - | - | - |
|  | Skeneidae | *Cirsonella romettensis* (Granata-Grillo, 1877) | Sr/M/F | U |  | U | - | - | - | - | - | L |
|  |  | cf. *Lissospira* sp. | Sr/M/F | U |  | U | - | - | - | - | R | - |
| **Bivalvia** |  |  |  |  |  |  |  |  |  |  |  |  |
| Unassigned | Unassigned | Bivalvia unassigned (juveniles) | U | U |  | U | U | - | - | U | - | - |
| Heterodonta | Cuspidariidae | *Cuspidaria sp.* | Sr/D/F | Ca/Pr | zoo;mei | 9 | - | - | - | - | - | - |
|  | Pholadidae | ***Xylophaga dorsalis* (Turton, 1819)** | Sr/S/Z | Om/Hs;Su | pom;ter | 19 | **N** | - | **N** | **N** | **N** | - |
|  |  | ***Xyloredo* sp.** | Sr/S/Z | Om/Hs;Su | pom;ter | 19 | - | - | - | **N** | - | - |
|  | Kelliellidae | *Kelliella* sp. (juveniles) | Ss/D/F | Om/De;Su | sed;pom;mic | 13 | L | - | - | - | - | - |
| Protobranchia | Neilonellidae | *Neilonella latior* (Jeffreys, 1876) | Ss/D/F | Om/De | sed;mic | 13 | - | - | - | - | - | - |
|  | Yoldiidae | *Yoldiella* sp.(juveniles) | Ss/D/B | Om/De | sed;pom;mic | 13 | - | - | - | - | R | - |
|  | Nuculidae | *Ennucula bushae* (Dollfus, 1898) | Ss/M/F | Om/De | sed;pom;mic | 13 | R | - | - | - | - | - |
|  | Solemyidae | Solemyidae (juveniles) | Ss/D/F | Ch | - | 20 | L | L | L | L | L | - |
| Pteriomorphia | Mytilidae | ***Idas modiolaeformis* (Sturany, 1896**) | Ep/D/A | Om/Ch;Su | pom | 20 | **N** | **N** | - | **N** | **N** | - |
|  | Pectinidae | *Delectopecten vitreus* (Gmelin, 1791) | Ep/D/F | Om/Su | pom | 16 | - | - | - | - | - | - |
|  | Propeamussidae | *Propeamussium* sp. | Ep/D/F | Om/Su | pom | 16 | - | - | - | - | - | - |
| **ARTHROPODA** |  |  |  |  |  |  |  |  |  |  |  |  |
| **Maxillopoda** |  |  |  |  |  |  |  |  |  |  |  |  |
| Cirripedia | Verrucidae | *Verruca* sp. | Ep/S/R | Om/Su | pom | 16 | - | - | - | - | - | - |
| **Malacostraca** |  |  |  |  |  |  |  |  |  |  |  |  |
| Decapoda | Unassigned | Dendrobrachiata unassigned | Sr/M/F | Om/Dt;Pr;Sc | pom;mic | 2 | - | - | - | - | - | - |
|  | Alpheidae | *Alpheus* sp. | Sr/M/F | Om/Dt;Pr;Sc | pom;mic;mei | 2 | - | - | - | - | - | - |
|  | Stenopodidae | *Richardina sp.* | Sr/M/F | Om/Dt;Pr;Sc | pom;mic,mei | 2 | - | - | - | - | - | - |
|  | Leucosiidae | *Ebalia nux* A. Milne-Edwards, 1883 | Sr/M/F | Om/Dt;Pr;Sc | pom;mic;mei | 2 | - | - | - | - | - | - |
|  | Xanthidae | *Monodaeus couchii* (Couch, 1851) | Sr/M/F | Om/Dt;Pr;Sc | pom;mic;mei | 2 | - | L | L | - | - | - |
| Euphausiacea |  | *Meganyctiphanes* cf.*norvegica* (M. Sars, 1857) | Ep/M/F | U |  | U | - | - | - | - | - | - |
| Nebaliacea |  | *Nebalia* spA | Sr/M/F | Ca/Sc | mac | 4 | L | L | - | - | L | - |
|  |  | *Nebalia* spB | Sr/M/F | Ca/Sc | mac | 4 | L | L | - | - | - | - |
| Amphipoda | Unassigned | Amphipoda unassigned | U | U |  | U | - | - | - | - | - | - |
|  | Aoridae | Aoridae unassigned | Sr/D/T | Om/Su | pom;mic | 15 |  |  |  |  |  |  |
|  | Calliopidae | ***Leptamphopus* sp122** | Ep/M/F | Ca/Pr | zoo | 5 | - | **N** | - | - | - | - |
|  |  | ***Leptamphopus* sp123** | Ep/M/F | Ca/Pr | zoo | 5 | - | - | - | - | **N** | - |
|  | Caprellidae | *Phtisica* sp. nov.? | Sr/M/F | Mc/Gr | mic | 10 | - | - | - | - | - | - |
|  | Dulichiidae | *Dulichiopsis nordlandica* (Boeck, 1870) | Sr/M/T | Mc/Gr | mic | 10 | - | L | - | - | - | - |
|  | Eusiridae | *Eusirus longipes* Boeck, 1861 | Ep/M/F | Ca/Pr | zoo | 5 | L | - | - | - | - | - |
|  | Liljeborgiidae | *Idunella* sp. | U | U |  | U | - | - | - | - | - | - |
|  |  | *Liljeborgia cf. dellavalei* Stebbing, 1906 | U | U |  | U | - | - | - | - | - | - |
|  | Lysianassidae | Lysianassidae spD | Sr/M/F | Ca/Sc | mac | 4 | - | - | - | - | - | U |
|  |  | ***Ensayara* cf. *carpinei* Bellan-Santini, 1974** | Sr/M/F | Om/Dt;Pr;Sc | mac | 3 | **N** | **N** | - | - | - | - |
|  |  | *Normanion ruffoi* Diviacco & Vader, 1988 | Sr/M/F | Om/Dt;Pr;Sc | mac | 3 | - | - | - | - | - | - |
|  |  | ***Orchomene grimaldii* Chevreux, 1890** | Sr/M/F | Om/Dt;Pr;Sc | mac | 3 | **N** | **N** | - | - | **N** | - |
|  |  | *Tryphosella simillima* Ruffo, 1985 | Sr/M/F | Ca/Sc | mac | 4 | - | R | - | - | - | - |
|  | Melitidae | *Eriopisa elongata* (Bruzelius, 1859) | Sr/M/F | Om/Dt | pom | 12 | - | - | - | - | - | - |
|  | Melphidippidae | *Melphidippella macra* (Norman, 1869) | Ep/M/F | Om/Su | pom;zoo | 16 | - | - | - | - | - | - |
|  | Oedicerotidae | Oedicerotidae unassigned | Ss/M/F | Ca/Pr | mei | 8 | - | - | - | - | - | - |
|  | Pardaliscidae | Pardaliscidae unassigned | Ep/M/F | Ca/Pr | zoo | 5 | - | - | - | - | - | - |
|  |  | *Pardaliscoides* sp. | Ep/M/F | Ca/Pr | zoo | 5 | - | - | - | - | - | - |
|  | Photidae | *Photis longicaudata* (Bate & Westwood, 1862) | Sr/D/T | Om/Su | pom;mic | 15 | - | - | L | - | - | - |
|  | Phoxocephalidae | *Harpinia* sp. | Sr/M/B | Ca/Pr | mei | 9 | - | L | - | - | - | - |
|  | [Phrosinidae](http://www.marinespecies.org/aphia.php?p=taxdetails&id=101421) | *Primno macropa* Guérin-Méneville, 1836 | Ep/M/F | U |  | U | - | - | - | - | - | - |
|  | Pleustidae | Pleustidae spA | Sr/M/F | Ca/Pr | mac | 7 | U | U | - | - | - | - |
|  |  | *cf. Pleusymtes mediterraneus* (Ledoyer, 1986) | Sr/M/F | Ca/Pr | mac | 7 | - | - | - | - | - | - |
|  | Sebidae | *Seba aloe* Karaman, 1971 | Sr/M/F | Mc/Gr | mic | 10 | - | - | - | L | L | - |
|  | Stenothoidae | *Stenothoe* sp. | Sr/M/F | Ca/Pr | mac | 7 | - | - | - | - | - | - |
| Cumacea | Leuconidae | *Leucon* sp. | Sr/M/F | Mc/Gr | mic | 10 | - | - | - | - | - | - |
|  | Nannastacidae | Nannastacidae unassigned | Ep/M/F | Om/Su | pom | 16 | - | - | - | - | - | - |
| Isopoda | Desmossomatidae | Desmossomatidae unassigned | Ss/M/F | Om/Dt | pom;mic | 1 | - | - | - | - | - | - |
|  |  | *Chelator* sp. | Ss/M/F | Om/Dt | pom;mic | 1 | - | - | - | - | R | - |
|  |  | *Prochelator* sp. | Ss/M/F | Om/Dt | pom;mic | 1 | - | - | - | - | R | - |
|  | Gnathiidae | *Gnathia* sp. | Ep/M/X | Ca/Sp | fis | 18 | L | L | L | - | - | - |
|  | Janiridae | *Austrofilius* cf. *mediterraneus* Castello, 2002 | Sr/M/F | Om/Dt | pom;mic | 12 | - | - | - | - | L | - |
|  |  | *Janira maculosa* Leach, 1814 | Sr/M/F | Om/Dt | pom | 12 | - | - | - | R | R | - |
|  | Munnidae | *Munna* sp. | Sr/M/F | Om/Dt | pom | 12 | L | - | L | L | - | - |
|  | Munnopsidae | *Disconectes* sp. | Sr/M/F | Om/Dt | pom;mic | 12 | - | - | - | - | - | - |
|  |  | *Ilyarachna* sp. | Sr/M/F | Om/Dt | pom;mic | 12 | - | - | - | - | - | - |
| Mysida |  | Mysida unassigned | U | U |  | U | - | - | - | - | - | - |
| Tanaidacea | Apseudidae | *Apseudes setiferus* Bacescu, 1981 | Sr/D/F | Om/Dt | pom | 12 | R | R | - | - | - | - |
|  | Unassigned | Tanaidomorpha unassigned | Sr/D/T | Om/Dt | pom | 12 | - | - | - | - | - | - |
|  | Leptocheliidae | *Mesotanais pinguiculus* Blazewicz-Paszkowycz, Bamber & Cunha, 2011 | Sr/D/T | Om/Dt | pom | 12 | - | - | - | L | L | L |
|  | Leptognathiidae | *Leptognathia* sp. | Sr/D/T | Om/Dt | pom | 12 | - | - | - | - | - | - |
|  | Pseudotanaidae | *Pseudotanais tympanobaculum* Błażewicz-Paszkowycz, Bamber & Cunha, 2011 | Sr/D/T | Om/Dt | pom | 12 | - | - | - | - | - | - |
|  | Tanaellidae | *Araphura macrobelone* Błażewicz-Paszkowycz, Bamber & Cunha, 2011 | Sr/D/T | Om/Dt | pom | 12 | - | - | - | - | - | - |
|  |  | *Tanaella unguicillata* Norman & Stebbing, 1886 | Sr/D/T | Om/Dt | pom | 12 | - | - | - | - | - | - |
| **ECHINODERMATA** | |  |  |  |  |  |  |  |  |  |  |  |
| **Ophiuroidea** | Unassigned | Ophiurida unassigned (juv.) | Sr/D/F | Om/Su;De | pom;mic;zoo | 15 | R | R | R | R | R | R |
| **Crinoidea** | Unassigned | Crinoidea unassigned (juv.) | Sr/S/A | U |  | U | - | - | - | - | - | U |
| **BRYOZOA** |  |  |  |  |  |  |  |  |  |  |  |  |
| Unassigned | Unassigned | Bryozoa unassigned | Ep/S/R | Om/Su | pom | 16 | - | U | - | - | - | - |

**Feeding habit**. Source of food: epibenthic (Ep); seafloor surface (Sr); subsurface (Ss). Motility: mobile (M); discretely motile, movement not necessary for feeding (D); sessile (S). Habit: free living or active burrower (F); tubiculous (T); sedentary, living in burrow (B); encrusting, requiring large area of attachment (R); attached, requiring one point of attachment (A); parasitic (X); unassigned (U). **Feeding mode**. Type of symbiosis: chemotophic (Ch); heterotrophic (Hs). Diet: carnivorous (Ca), omnivorous (Om), feeding on microbes (Mc). Mode: deposit feeder, ingest sediment (De); detritus feeder, ingests particulate matter (Dt); suspension, filter feeder , strains particles from the water (Su); grazer, feeds by scraping (Gr); predator, eats living animals (Pr); scavenger, eats carrion (Sc); suctorial parasite (Sp); symbiotic (Sym); unassigned (U). **Food type and size**. Sediment (sed), particulate organic matter (pom); wood and other terrestrial or coastal plant material (ter); single celled organisms (mic); meiofauna (mei); macrofauna (mac); zooplankton (zoo); fish (fis); unassigned (U). The trophic scheme is based on Macdonald et al. [1]. Trophic information was obtained from stable isotope data (mostly unpublished) and from the literature for each individual species wherever possible; if the feeding behaviour of a particular species was unknown, it was assumed to feed in a similar manner to congeneric or confamilial species, or species within the same major group.

**Trophic guilds.** For simplification we established 20 different trophic guilds based on source of food, feeding mode and diet: 1. Omnivores on subsurface items; 2. Omnivores on surface small items; 3. Omnivores on surface large items; 4. Scavengers; 5. Predators on zooplankton; 6. Predators on subsurface macrofauna; 7. Predators on surface macrofauna; 8. Predators on subsurface meiofauna; 9. Predators on surface meiofauna; 10. Microbial grazers; 11. Microbial grazers, wood specialists; 12. Detritivores; 13. Subsurface deposit feeders; 14. Surface deposit feeders; 15. Suspension feeders on surface items; 16. Suspension feeders on epibenthic items; 17. Suctorial parasites on macrofauna; 18. Suctorial parasites on fish; 19. Heterotrophic symbiosis; 20. Chemotrophic symbiosis; U. unassigned.

**Sub-regions:** El Arraiche, Mercator MV (EA); Carbonate Province, Meknès and Darwin MV (CP). **Type of substrate:** wood (W); alfalfa (A); carbonate (C). **Occurrence**: new occurrences (N); taxa previously recorded in the region (R); taxa previously recorded in the study site (L); unassigned taxa (U).

**Reference:**

1. Macdonald TA, Burd BJ, Macdonald VI, van Roodselaar A (2010) Taxonomic and feeding guild classification for the marine benthic macroinvertebrates of the Strait of Georgia, British Columbia. Can Tech Rep Fish Aquat Sci 2874: iv + 63 p.
