# Supplementary material for: Are Organic Falls Bridging Reduced Environments in the Deep Sea? - Results from Colonization Experiments in the Gulf of Cádiz
Source: PLoS One. 2013 Oct 2;8(10):e76688. doi: 10.1371/journal.pone.0076688 (PMC3788751; doi:10.1371/journal.pone.0076688)
Supplement: Table S2 — Breakdown of percentual contributions from SIMPER analysis for comparisons between mud volcanoes: Mercator (Mer); Meknès (Mek) and Darwin (Dar). The taxa listed contribute at least 1.5%. Numbers in bold mark the six dominant species at each site. (DOC) [file pone.0076688.s004.doc]

Table S2. Breakdown of percentual contributions from SIMPER analysis for comparisons between mud volcanoes: Mercator (Mer); Meknès (Mek) and Darwin (Dar). The taxa listed contribute at least 1.5%. Numbers in bold mark the six dominant species at each site.

|  |  | Abundance (ind.m^-2^) | | |  | % Contribution | | |  | % Contribution | | |
| --- | --- | --- | --- | --- | --- | --- | --- | --- | --- | --- | --- | --- |
|  |  | Mer | Mek | Dar | TG | Mer | Mek | Dar |  | Mer/Mek | Mer/Dar | Mek/Dar |
|  | Total | 654.1 | 637.6 | 886.0 |  | AS:18.4 | AS:20.6 | AS:28.7 |  | AD:92.4 | AD:91.6 | AD:71.2 |
| **Cnidaria** |  |  |  |  |  |  |  |  |  |  |  |  |
| Hydrozoa | Hydrozoa und | 1.0 | 0.0 | 0.7 | E-P | 7.5 | --- | ● |  | 1.5 | ● | ● |
|  | *Clytia linearis* | 0.7 | 0.3 | 0.0 | E-P | 3.7 | ● | --- |  | ● | ● | ● |
| **Sipuncula** |  |  |  |  |  |  |  |  |  |  |  |  |
| Sipuncula und. |  | 0.5 | 0.0 | 0.0 | S-Dt | 3.1 | --- | --- |  | ● | ● | --- |
| **Nemertea** |  |  |  |  |  |  |  |  |  |  |  |  |
| Nemertea und. |  | 0.2 | 0.3 | 0.7 | S-P | ● | ● | 1.7 |  | ● | ● | ● |
| **Annelida** |  |  |  |  |  |  |  |  |  |  |  |  |
| incertae sedis | *Protodrilus* sp. | 0.0 | 0.0 | 10.3 | S-Gr | --- | --- | 2.4 |  | --- | ● | 1.8 |
| Aciculata | *Ophryotrocha* sp01 | 15.0 | 1.3 | 0.0 | S-O | ● | ● | --- |  | 1.5 | ● | ● |
|  | *Ophryotrocha* sp02 | **16.2** | 4.0 | 4.3 | S-O | 1.7 | 3.3 | ● |  | 1.9 | 1.5 | 1.5 |
|  | *Ophryotrocha* sp08 | 0.0 | **14.3** | 8.0 | S-O | --- | 3.7 | 2.5 |  | 2.0 | ● | 2.3 |
|  | *Leocrates atlanticus* | 0.5 | 1.0 | 0.3 | S-P | ● | 8.3 | ● |  | 1.5 | ● | ● |
|  | *Nereimyra* sp. | 2.2 | 1.3 | 12.3 | S-P | ● | 2.3 | 3.6 |  | ● | 1.7 | 2.2 |
|  | *Harmothoe evei* | 5.0 | 0.3 | 0.0 | S-P | 18.3 | ● | --- |  | 2.7 | 2.1 | ● |
|  | *Subadyte pelucida* | 0.7 | 0.3 | 1.3 | S-P | 3.7 | ● | ● |  | ● | ● | ● |
|  | Exogoninae sp. | 0.2 | 0.0 | 7.3 | S-Gr | ● | --- | 1.7 |  | ● | ● | 1.5 |
| Canalipalpata | *Amage* sp. | 0.2 | 1.7 | 42.7 | S-De | ● | ● | 5.1 |  | ● | 3.1 | 3.9 |
|  | *Mellinopsis* sp. | 1.8 | **49.0** | **79.3** | S-De | ● | 8.1 | 7.4 |  | 4.0 | 4.4 | 5.7 |
|  | *Raricirrus beryli* | 9.0 | 4.3 | 24.3 | S-Gr | ● | ● | 4.6 |  | 1.7 | 2.5 | 3.0 |
|  | *Polycirrus norvegicus* | 3.3 | 0.0 | 0.0 | S-De | 4.8 | --- | --- |  | ● | ● | --- |
| Scolecida | Capitellidae sp05 | 1.0 | 0.0 | 0.0 | S-De | 5.7 | --- | --- |  | 1.6 | ● | --- |
| **Mollusca** |  |  |  |  |  |  |  |  |  |  |  |  |
| Heterodonta | *Xylophaga dorsalis* | **362.3** | 3.7 | 3.7 | Sy | 14.1 | 1.7 | ● |  | 8.9 | 7.5 | ● |
| Protobranchia | Solemyidae juv. | 0.7 | 4.3 | 0.7 | Sy | 1.8 | ● | ● |  | ● | ● | ● |
| Pteriomorphia | *Idas modiolaeformis* | 0.3 | **101.7** | **106.0** | Sy | ● | 14.5 | 9.5 |  | 5.8 | 4.9 | 6.8 |
| Caenogastropoda | Eulimidae sp01 | 0.0 | 0.0 | 0.7 | S-Sp | --- | --- | ● |  | --- | ● | ● |
| Cocculiniformia | *Coccopigya* sp. | 0.5 | 7.7 | 57.0 | S-Gr | ● | 4.4 | 3.7 |  | 1.6 | 3.1 | 3.9 |
| Heterobranchia | *Xylodiscula* sp. | 0.5 | 1.7 | 31.3 | S-Gr | ● | 2.3 | 2.2 |  | ● | 2.2 | 2.8 |
| Vetigastropoda | *Copulabyssia* sp. | 3.2 | **253.7** | **179.7** | S-Gr | ● | 26.3 | 13.7 |  | 9.4 | 6.4 | 9.8 |
| **Arthropoda** |  |  |  |  |  |  |  |  |  |  |  |  |
| Amphipoda | *Leptamphopus* sp122 | **26.8** | 0.0 | 0.0 | E-P | ● | --- | --- |  | 2.7 | 1.9 | --- |
|  | *Orchomene grimaldii* | **147.2** | 0.7 | 0.0 | S-O | 5.9 | ● | --- |  | 5.7 | 4.7 | ● |
|  | *Seba aloe* | 0.0 | **153.7** | **213.0** | S-Gr | --- | 20.7 | 10.9 |  | 7.4 | 6.6 | 9.0 |
| Isopoda | *Gnathia* sp. | **10.2** | 0.0 | 0.0 | E-Sp | 6.5 | --- | --- |  | 3.0 | 2.2 | --- |
|  | *Janira maculosa* | 0.0 | 1.7 | 0.0 | S-Dt | --- | 1.7 | --- |  | ● | --- | ● |
|  | *Munna* sp. | 0.8 | 1.3 | 0.3 | S-Dt | 1.6 | ● | ● |  | ● | ● | ● |
| Tanaidacea | *Apseudes setiferus* | 1.3 | 0.0 | 0.0 | S-Dt | 1.9 | --- | --- |  | ● | ● | --- |
|  | *Mesotanais pinguiculus* | 0.0 | 3.7 | **59.7** | S-Dt | --- | 2.9 | 13.5 |  | ● | 5.1 | 5.7 |
| **Echinodermata** |  |  |  |  |  |  |  |  |  |  |  |  |
| Ophiurida | Ophiurida juv. | 0.7 | 0.3 | 24.3 | S-Su | 3.7 | ● | 10.3 |  | ● | 3.1 | 4.8 |
|  |  |  |  |  |  |  |  |  |  |  |  |  |
| % Contribution of selected taxa | | 93.5 | 92.5 | 93.8 |  | 87.8 | 100.0 | 96.3 |  | 74.9 | 76.8 | 75.7 |

TG: trophic guild; AS: average similarity; AD: Average dissimilarity; E: epibenthic source of food; S: sediment surface or subsurface source of food; De: deposit feeder; Dt: detritus feeder; Gr: Grazer; O: Omnivore; P: predator; Sp: suctorial parasite; Su: suspension feeder; Sy: symbiotic; ●: contributions lower than 1.5%.
