# Supplementary material for: Are Organic Falls Bridging Reduced Environments in the Deep Sea? - Results from Colonization Experiments in the Gulf of Cádiz
Source: PLoS One. 2013 Oct 2;8(10):e76688. doi: 10.1371/journal.pone.0076688 (PMC3788751; doi:10.1371/journal.pone.0076688)
Supplement: Table S3 — Breakdown of percentual contributions from SIMPER analysis for comparisons between substrate types: wood (W), alfalfa (A) and carbonate (C). The taxa listed contribute at least 1.5%. Numbers in bold mark the six dominant species in each substrate type. (DOC) [file pone.0076688.s005.doc]

Table S3. Breakdown of percentual contributions from SIMPER analysis for comparisons between substrate types: wood (W), alfalfa (A) and carbonate (C). The taxa listed contribute at least 1.5%. Numbers in bold mark the six dominant species in each substrate type.

|  |  | Density (ind.m^-2^) | | |  | % Contribution | | |  | % Contribution | | |
| --- | --- | --- | --- | --- | --- | --- | --- | --- | --- | --- | --- | --- |
|  |  | W | A | C | TG | W | A | C |  | W/A | W/C | A/C |
|  | Total | 1302.8 | 786.3 | 35.0 |  | AS:29.6 | AS:15.6 | AS:9.8 |  | AD:74.3 | AD:93.3 | AD:92.5 |
| **Cnidaria** |  |  |  |  |  |  |  |  |  |  |  |  |
| Hydrozoa | Hydrozoa und | 0.3 | 0.5 | **1.3** | E-P | ● | ● | 31.3 |  | ● | ● | ● |
|  | *Clytia linearis* | 0.5 | 0.3 | 0.5 | E-P | ● | ● | 9.2 |  | ● | ● | ● |
| **Nemertea** |  |  |  |  |  |  |  |  |  |  |  |  |
| Nemertea und. |  | 0.3 | 0.0 | 0.5 | S-P | ● | --- | 8.4 |  | ● | ● | ● |
| **Annelida** |  |  |  |  |  |  |  |  |  |  |  |  |
| Aciculata | *Ophryotrocha* sp01 | 23.5 | 0.0 | 0.0 | S-O | 3.7 | --- | --- |  | 2.6 | 2.9 | --- |
|  | *Ophryotrocha* sp02 | 25.8 | 4.8 | 0.0 | S-O | 2.8 | 5.3 | --- |  | 2.1 | 2.8 | 1.6 |
|  | *Ophryotrocha* sp08 | 11.8 | 5.0 | 0.0 | S-O | ● | 2.1 | --- |  | 1.7 | 1.8 | ● |
|  | cf. *Amphiduros* sp. | 2.8 | 1.3 | 0.0 | S-Sc | 1.7 | 4.0 | --- |  | ● | ● | ● |
|  | *Leocrates atlanticus* | 1.0 | 0.3 | 0.5 | S-P | ● | ● | 15.8 |  | ● | ● | ● |
|  | *Nereimyra* sp. | 8.5 | 5.0 | 0.0 | S-P | 3.0 | ● | --- |  | 1.5 | 1.9 | ● |
|  | *Harmothoe evei* | 3.5 | 2.8 | **1.5** | S-P | 1.9 | 4.4 | 8.4 |  | ● | ● | 2.1 |
|  | *Subadyte pelucida* | 0.6 | 1.3 | 0.3 | S-P | 1.5 | 4.1 | ● |  | ● | ● | ● |
| Canalipalpata | *Amage* sp. | 24.5 | 9.0 | 0.0 | S-De | 2.0 | ● | --- |  | 2.4 | 2.6 | ● |
|  | *Mellinopsis* sp. | **72.3** | 26.8 | 0.0 | S-De | 7.1 | 13.0 | --- |  | 4.1 | 5.2 | 4.6 |
|  | *Raricirrus beryli* | 20.8 | 14.3 | 0.0 | S-Gr | 2.9 | 3.3 | --- |  | 2.50 | 3.0 | 2.1 |
|  | *Polycirrus norvegicus* | 3.8 | 0.5 | 0.8 | S-De | ● | 2.2 | ● |  | ● | ● | ● |
| Scolecida | Capitellidae sp05 | 0.0 | 0.3 | **1.3** | S-De | --- | ● | 11.8 |  | ● | ● | ● |
| **Mollusca** |  |  |  |  |  |  |  |  |  |  |  |  |
| Heterodonta | *Xylophaga dorsalis* | **543.3** | 0.8 | 5.0 | Sy | 22.2 | ● | ● |  | 13.2 | 15.5 | 1.5 |
| Pteriomorphia | *Idas modiolaeformis* | **88.8** | **67.5** | 0.0 | Sy | 6.2 | 10.5 | --- |  | 4.8 | 5.5 | 5.2 |
| Cocculiniformia | *Coccopigya* sp. | 9.5 | **39.8** | 0.0 | S-Gr | 3.8 | 2.4 | --- |  | 2.6 | 2.1 | 2.8 |
| Heterobranchia | *Xylodiscula* sp. | 2.8 | 22.8 | 0.0 | S-Gr | 2.0 | 4.0 | --- |  | 1.6 | ● | 2.3 |
| Vetigastropoda | *Copulabyssia* sp. | **200.5** | **129.3** | 0.0 | S-Gr | 11.8 | 17.1 | --- |  | 7.1 | 8.6 | 7.7 |
| **Arthropoda** |  |  |  |  |  |  |  |  |  |  |  |  |
| Leptostraca | *Nebalia* sp01 | 1.5 | 2.5 | 0.0 | S-Sc | ● | 4.7 | --- |  | ● | ● | 1.5 |
| Amphipoda | *Ensayara carpinei* | 1.0 | 13.3 | 0.0 | S-O | ● | ● | --- |  | 1.5 | ● | 2.4 |
|  | *Leptamphopus* sp122 | 0.0 | **40.3** | 0.0 | E-P | --- | 2.2 | --- |  | 2.6 | --- | 4.8 |
|  | *Orchomene grimaldii* | **61.8** | **159.5** | 0.0 | S-O | ● | 2.1 | --- |  | 6.1 | 3.0 | 8.6 |
|  | *Seba aloe* | **117.3** | **157.8** | 0.0 | S-Gr | 6.08 | 11.6 | --- |  | 6.3 | 6.1 | 7.2 |
| Isopoda | *Gnathia* sp. | 1.0 | 1.3 | **13.0** | E-Sp | ● | ● | 8.4 |  | ● | 1.7 | 2.6 |
|  | *Munna* sp. | 2.3 | 0.0 | 0.3 | S-Dt | 3.37 | --- | ● |  | ● | ● | ● |
| Tanaidacea | *Mesotanais pinguiculus* | 10.8 | 35.0 | **1.8** | S-Dt | ● | 2.6 | ● |  | 2.5 | 1.7 | 3.0 |
| **Echinodermata** |  |  |  |  |  |  |  |  |  |  |  |  |
| Ophiurida | Ophiurida juv. | 3.5 | 14.3 | **1.8** | S-Su | 2.98 | ● | 6.8 |  | ● | ● | 2.2 |
|  |  |  |  |  |  |  |  |  |  |  |  |  |
| % Contribution of selected taxa | | 95.4 | 96.1 | 80.7 |  | 88.1 | 99.1 | 100.0 |  | 73.9 | 74.9 | 73.5 |

TG: trophic guild; AS: average similarity; AD: Average dissimilarity; E: epibenthic source of food; S: sediment surface or subsurface source of food; De: deposit feeder; Dt: detritus feeder; Gr: Grazer; O: Omnivore; P: predator; Sc: scavenger; Sp: suctorial parasite; Su: suspension feeder; Sy: symbiotic; ●: contributions lower than 1.5%.
